# Risk of Alzheimer’s Disease in Cancer Patients: Analysis of Mortality Data from the US SEER Population-Based Registries

**DOI:** 10.3390/cancers12040796

**Published:** 2020-03-26

**Authors:** Roman Mezencev, Yury O. Chernoff

**Affiliations:** 1 School of Biological Sciences, Georgia Institute of Technology, Krone Engineered Biosystems Building, 950 Atlantic Drive NW, Atlanta, GA 30332-2000, USA; 2 Laboratory of Amyloid Biology, St. Petersburg State University, St. Petersburg 199034, Russia

**Keywords:** Alzheimer’s disease, Early-Onset Alzheimer’s disease, Late-Onset Alzheimer’s disease, cancer, BRCA1, SEER, chemotherapy, radiotherapy, mortality

## Abstract

Previous studies have reported an inverse association between cancer and Alzheimer’s disease (AD), which are leading causes of human morbidity and mortality. We analyzed the SEER (Surveillance, Epidemiology, and End Results) data to estimate the risk of AD death in (i) cancer patients relative to reference populations stratified on demographic and clinical variables, and (ii) female breast cancer (BC) patients treated with chemotherapy or radiotherapy, relative to those with no/unknown treatment status. Our results demonstrate the impact of race, cancer type, age and time since cancer diagnosis on the risk of AD death in cancer patients. While the risk of AD death was decreased in white patients diagnosed with various cancers at 45 or more years of age, it was increased in black patients diagnosed with cancers before 45 years of age (likely due to early onset AD). Chemotherapy decreased the risk of AD death in white women diagnosed with BC at the age of 65 or more, however radiotherapy displayed a more complex pattern with early decrease and late increase in the risk of AD death during a prolonged time interval after the treatment. Our data point to links between molecular mechanisms involved in cancer and AD, and to the potential applicability of some anti-cancer treatments against AD.

## 1. Introduction

Alzheimer’s disease (AD) and cancer are widespread diseases that have become major public health problems and leading causes of morbidity and mortality around the world. Cancer represents the second leading cause of death in the United States (US), with almost 1,700,000 new cases and 600,000 cancer-related deaths estimated for the year of 2016 [1]. AD is reportedly the sixth leading cause of death in the United States [2], with 5,000,000 estimated prevalent cases [3] and 93,541 reported deaths [2] in 2014, however recent publications indicate that this is an underestimate and evaluate AD as the third leading cause of death in the US [4], and possibly in other developed countries with the longest life expectancies. At a molecular level, AD is associated with accumulation of misfolded polymerized proteins, amyloid β (Aβ) and tau, in brains, followed by neuronal degeneration, dementia and eventually, death. Only a small fraction of AD cases are caused by autosomal dominant AD mutations, usually in the gene coding for the Aβ precursor or for the proteins that control processing of Aβ from the precursor. The majority of AD cases are of sporadic nature and are typically detected in older individuals (over 65, with a steep increase associated with further aging) [5,6]. 

Comorbidity (a term coined by Feinstein in his seminal paper [7]) has been recognized as a critically important concept with implications in clinical medicine, public health and health care planning, but also for elucidation of disease etiology and effects of therapeutic interventions [8]. Analysis of patterns of co-occurrence of complex diseases [9] or complex and Mendelian diseases [10] has potential to identify genuine comorbidity (or inverse comorbidity) for diseases that co-occur in individual patients significantly more frequently (or less frequently) than it would be expected by chance. Positive or inverse associations between diseases may reflect (i) the existence of shared genetic and/or environmental risk factors, (ii) effects of treatment, (iii) the role of a third disease with its own genetic or environmental risk factors that influences the occurrence of both diseases, or (iv) phenotypic causality, in which one disease is a direct cause of the other disease (directional causation), or both disorders may cause one another (reciprocal causation) [11].

Although both AD and cancer share several common risk factors, such as older age [6,12], obesity [6,13], type 2 diabetes [6,14] and smoking [6,15,16], inverse co-morbidity has been consistently found for these two diseases by observational epidemiological studies [17]. The inverse association between AD and cancer was first suggested from findings of autopsy-based cross-sectional studies, which identified lower prevalence of incidental cancers in patients with AD, as compared to institutionalized patients with other mental health conditions [18,19]. These intriguing findings called for further investigation of the role of biological, demographic (e.g., age) and lifestyle factors (e.g., smoking) that could provide adequate explanations [19]. 

These findings were also consistent with anecdotal observations, which noticed that the nursing home residents with dementia (probably caused by AD, as it is responsible for the majority of the cases of aging-associated dementia) have less likely been previously diagnosed with cancer than residents without dementia [20]. The inverse association between cancer and AD was further supported by cross-sectional studies, which reported a lower proportion of AD patients undergoing current or past treatment for cancer as compared to matched non-AD populations [21], or cohort studies that evaluated AD prevalence [22] or mortality [23,24]. More recently, carefully designed cohort studies reported a bi-directional inverse association between AD and cancer with striking consistency (reviewed in Reference [25]). A meta-analysis of cohort and nested case-control studies published up to July 2013 reported a significantly decreased risk of cancer in individuals with AD (effect size, ES = 0.50; 95% confidence interval, CI95 = 0.34−0.74) and a significantly decreased risk of AD in individuals diagnosed with cancers (ES = 0.64; CI95 = 0.56−0.73) [26]. Similarly, a meta-analysis of cohort and case-control studies published in May 2014 reported a pooled estimate of relative risk of cancer in AD individuals as 0.55 (CI95 = 0.41−0.75) [27]. Consistency across studies, bidirectionality of the inverse association between AD and cancer and the absence of an inverse association between vascular dementias and cancers further support the strength of epidemiological evidence of inverse co-morbidity between these two complex diseases. 

In this study, we investigated the association between various solid tumors and leukemias and death due to AD, using the data reported by population-based SEER (Surveillance, Epidemiology, and End Results) Cancer Registries, which is considered the gold standard among cancer registries worldwide [28]. The risk of AD-specific mortality is compared (i) between cancer patients and the general population and (ii) among women diagnosed with female breast cancer to assess the influence of demographic and clinical variables on the risk of death due to AD. Our results indicate that the association between cancer and AD depend on the race and age at cancer diagnosis. The analysis of SEER registry data supports a decreased risk of AD death in white patients, diagnosed with cancer at the age of ≥45 years. We confirm this protective effect with several solid tumors and hematopoietic malignancies and show that its magnitude is modified by the age at which cancer is diagnosed. Importantly, our data show that the risk of AD death is influenced by therapeutic treatments applied to cancer patients. While chemotherapy is associated with a significant decrease in the risk of AD-related death in breast cancer patients, radiotherapy shows a more complex pattern with early decrease and delayed increase in the risk of AD death in these individuals. 

## 2. Results

### 2.1. Impact of Age at Diagnosis and Race on the Risk of AD Death in Cancer Patients

A total of 3,891,540 cancer patients were included in this study. Mean age at the time of cancer diagnosis was 64 years. During the follow-up time of 0–42 years (with a mean of 6.43 years), 23,166 patients died due to Alzheimer’s disease at a mean age of 87.40 years. Crude cause-specific death rate for the Alzheimer’s disease was 92.52 per 100,000 persons per year (person-years). Cohort characteristics for race and age subgroups are presented in Table 1.

Analysis of the risk of AD death for cancer patients diagnosed before the age of 45 years (median: 36 years) produced only a limited set of informative results (Table S2). This was due to the relatively short follow-up times (median: 8.3 years) for these patients, that result in a relatively young age at the last follow-up time point for many of these patients (median: 44 years), meaning that 398,545 cases (equal to 92% of all cases in this age group) were followed only up to the age of below 65 years. Since 95% of AD cases are the late-onset AD (LOAD) cases, which by definition is diagnosed in patients 65+ years of age [5], the SEER data cannot provide sufficient information about the effect of cancer diagnosed at a young age on the risk of this (prevailing) type of AD. Indeed, a substantial proportion of AD deaths in this group occurred before the age of 74 years (53 cases of all 87 AD deaths), and a significant portion of them are likely attributable to the relatively rare (and usually heritable) early-onset type of AD (EOAD) [29], which typically represents only about 5% of all AD cases. As a result, these data do not allow for inferences about the risk of death due to late-onset AD, while the inferences about the risk of death from EOAD are affected by small numbers of cancer cases and AD death events.

White patients diagnosed with any cancer before the age of 45 years did not differ significantly in the risk of AD death from the reference population (Table S2). Among specific cancer sites with at least 100,000 accumulated person-years, only brain cancer patients diagnosed before the age of 45 displayed increased risk of AD death relative to the reference population (standardized mortality ratio (SMR) = 10.00; CI95: 2.06–29.22) (Table S2); however, this could be due to the small number of observed AD deaths (*N* = 3), and therefore, may represent a statistical fluctuation. 

Black patients diagnosed with any cancer before the age of 45 years displayed a significantly increased risk of AD death, compared to the reference population for all follow-up times combined (SMR = 2.28; CI95: 1.09–4.19). This increased risk is most likely attributable to the early-onset AD death, which is supported by the fact that 7 of the 10 recorded AD deaths occurred before the age of 70 years. However, a relatively small number of cases in combination with a relatively diverse range of primary cancers (Table S3) limit confidence and interpretability of this result. The SMR values for specific cancers were not significant, except for testicular cancer, which showed an extremely high SMR value, but in just one follow-up interval (Table S2) and on the basis of just a single AD fatality, making this result unreliable on its own. The only cancer type which occurred more than once among 10 cases of AD was female breast cancer (Table S3). While its SMR value suggested increased risk, the 95% confidence interval overlapped 1, making the association of female breast cancer diagnosed in young black women with increased risk of AD death inconclusive (SMR = 2.79; CI95: 0.76–7.14; *N* = 4 cases). 

American Indian/Alaska Native (AIAN) cancer patients diagnosed before the age of 45 years showed no significant SMR values for AD death. Asian/Pacific Islander (API) patients showed one significant SMR value for all sites combined in just one follow-up interval (Table S2), however this result could originate from a random fluctuation as it was based on a single AD fatality case.

Overall, our analysis shows that that SEER 9 registry data on patients diagnosed with cancers before the age of 45 years are not informative regarding the risk of death from LOAD (prevailing type of AD). Data in black patients younger than 45 years are suggestive of a possible correlation between cancer and EOAD, although the nature of this correlation still has to be determined. As EOAD is usually a heritable disease, cancer cannot increase the risk of EOAD in the person who already has a mutation. It is, however, possible that either black people with EOAD mutations have a higher risk of cancer development, or cancer speeds up EOAD progression leading to earlier death from EOAD. Possible reasons for this are considered in the Discussion Section. 

### 2.2. Decreased Risk of Death Due to AD in White Patients Diagnosed with Different Types of Cancer at the Age of ≥ 45 Years 

When all cancer sites were considered together, patients of white races, who received a cancer diagnosis at the age of ≥45 years, displayed significantly reduced risk of AD death relative to the reference population for follow-up intervals of 0–11 months, 12–59 months and 60–119 months (Table 2). However, their risk of AD death was higher than the risk in the reference population for follow-up times 120 or more months since diagnosis. Nevertheless, the risk of AD death at all follow-up times combined remained significantly reduced for these patients relative to the reference population (SMR = 0.97; CI95: 0.96–0.99; Table 2).

A similar pattern of the risk of AD death was observed in these patients across several specific cancer sites, which showed a decreased risk for one or more follow-up intervals within 119 months, but an increased risk of AD death at the follow-up period starting 120 months since cancer diagnosis. Nevertheless, the overall risk of AD death remained significantly decreased in patients diagnosed with lung cancer or urinary bladder cancer. 

A somewhat different pattern was found in patients diagnosed with cancers of the oral cavity, colon, rectum, female breast, corpus uteri, prostate and kidneys, who also displayed increased risk of AD death after 120 months, and reduced risk of AD death for earlier time intervals, but no overall reduction of the risk for all follow-up intervals combined. Patients with non-Hodgkin lymphomas, chronic lymphocytic leukemias and all lymphoproliferative and hematologic malignancies combined a displayed lower risk of AD death across all follow-up times, except 120+ months, when their risk of AD death was similar to that in the reference populations. 

Asian/Pacific Islander patients displayed a rather different pattern of the risk of AD death for all cancer sites combined, all solid tumors combined, colon cancer, prostate cancer and urinary bladder cancer. In all these cancers, the risk of AD death was significantly increased for all follow-up times combined (e.g., for all cancer sites SMR = 1.23; CI95: 1.15–1.31). Except for bladder cancer, the risk of AD death in the patients with these cancers was significantly increased for at least one of the follow-up intervals before 120 months since diagnosis. Therefore, cancer does not seem to provide API patients with such a level of protection against AD that would be comparable to the protection observed in the white patients.

The risk of AD death in black patients diagnosed with cancer at ≥45 years of age, relative to the reference population, appears to display a similar magnitude and trend across follow-up times as in white patients, but the lower number of person-years accumulated for this cohort is a likely cause for why the differences between cancer and reference populations are not statistically significant in this cohort. A cancer site showing apparently different association with AD death between black and white patients is the uterine cervix. SMR values for white women suggest a protective effect at 60–119 months (SMR = 0.48; CI95: 0.19–0.99; *N* = 7) and no difference from the reference population overall (SMR = 0.93; CI95: 0.76–1.13; *N* = 102), but for black women, the effect appears to be deleterious for both 60–119 months (SMR = 3.13; CI95: 1.35–6.16; *N* = 8) and overall (SMR = 1.56; CI95: 1.01–2.31; *N* = 25). The cohorts of black patients and white patients with cancers of the uterine cervix display differences in distributions of cancer histological types, stages and grades, but not in mean ages at cancer diagnosis, years of cancer diagnosis or proportions of patients receiving chemotherapy or radiotherapy that included beam radiation (Supplementary Materials, Section S2).

### 2.3. Protective Effect of Cancer Against Death from AD is Modified by Age 

**Figure 1:**
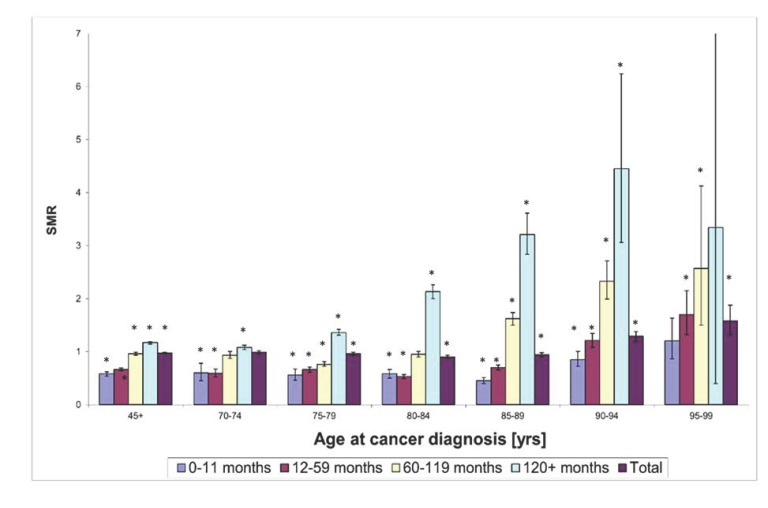
Standardized mortality ratios (SMRs) for death due to AD in white-race patients diagnosed with cancer at 45+ years of age. SMRs are stratified by 5-year age groups at cancer diagnosis and five follow-up intervals after cancer diagnosis. Error bars correspond to 95% confidence intervals (CI) for SMR (the upper-bound 95% CI for age group 95–99 years and latency 120+ months has a value of 12.05, which exceeds the maximum on the vertical scale); * *p* < 0.05 (not adjusted for multiple tests).

At a follow-up interval of 120 months and more, no type of cancer associated with a reduced risk of AD death was found in white patients (Table 2). This result, which is consistent across all cancer sites, suggests that either the protective effect of cancer in regard to AD-associated death is operating only within a limited timeframe from cancer diagnosis and therapeutic interventions, or the protection is lost with the further advance in age. The values of SMR for all cancer sites combined, determined for 5-year intervals for the age groups from 70 to 99 years, indeed indicate that cancers diagnosed at advanced ages are not associated with a decreased risk of AD death (Figure 1). To the contrary, patients diagnosed with cancer at the age of 90 to 94 years displayed a significantly increased risk of AD death relative to the reference population for each follow-up time interval beyond 11 months since cancer diagnosis, as well as for all follow-up time intervals combined (Figure 1). In the case of the oldest age group (95–99 years old at cancer diagnosis), even recently diagnosed cancers showed no protection from the AD death, which supports the age-dependence of the impact of cancer on the risk of AD death. Therefore, the loss of protection in the younger age groups for the follow-up intervals above 120 months is also likely related to the modification of the effect of cancer on the risk of AD death due to age. This interpretation is supported by the fact that white patients, who were diagnosed with cancer at 45+ years of age and survived for 120 or more months after the cancer diagnosis (*N* = 12,525), have a median age at AD death of 89 years, and 5481 of these patients (~44%) died of AD at the age of ≥90 years. Since the age groups 90–94 years and 95–99 years displayed an increased risk of death due to AD relative to the reference populations for several follow-up time intervals, a substantial proportion of cancer patients surviving 120 or more months attained ages at which the protective effect of cancer was reversed to a deleterious effect, which projected into the increased risk of AD death starting 120 months since the cancer diagnosis. 

**Figure 2:**
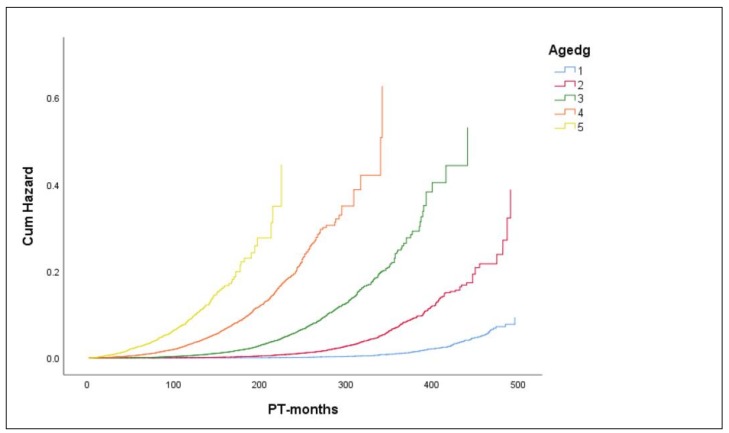
Baseline cumulative hazards of death from AD in patients diagnosed with breast cancer (BC). Data are shown for different time intervals after cancer diagnosis (PT-months), stratified by 5-year age groups at cancer diagnosis (Agedg) as follows: 1 (45–54 years), 2 (55–64 years), 3 (65–74 years), 4 (75–84 years) and 5 (85+ years).

The complex relationship between the risk of AD death, age at diagnosis and time since cancer diagnosis is demonstrated by baseline cumulative hazard functions for AD death. The baseline cumulative hazard functions, which were estimated by a stratified Cox proportional hazards model for breast cancer (BC) patients, suggest an increase in the risk of AD death with increasing age at BC diagnosis for the same timepoints since cancer diagnosis (Figure 2). This finding is not unexpected, considering that age at BC diagnosis plus time since BC diagnosis represents the attained age, and the AD mortality is well known to increase with age. However, this result also suggests that the effect of age at cancer diagnosis on the hazard of AD death is changing with time. To illustrate the non-proportionality of hazards between different age groups, we determined the point estimates of cumulative hazard ratios (cHR) between the age group 5 (85+ years) and the age group 3 (65–74 years) at different timepoints since BC diagnosis (Figure S1). The non-monotonous trend of cHR over time further supports a complex influence of age at diagnosis and time since cancer diagnosis on the risk of AD death, with maximum cHR reached within 30 months since diagnosis (cHR ~40), followed by a monotonous decrease to cHR ~12 at 178 months since diagnosis (Figure S1). We also found that age-specific AD mortality rates increased with increasing attained age for the fixed age groups at cancer diagnosis, which could result from possible effects of higher attained age or longer time intervals since cancer diagnosis, or both. However, age-specific mortality rates decreased with an increase in the age at cancer diagnosis at fixed attained ages, indicating an increased risk of AD death with increasing follow-up intervals (Supplementary Materials, Section S3). We hypothesize that this finding is explained by the reduction of the protective effect of cancer or interventions associated with cancer therapy over time since cancer diagnosis.

### 2.4. Chemotherapy and Radiation Therapy Influence the Risk of AD Death in Breast Cancer Patients 

#### 2.4.1. Analysis of SEER 13 Registry Data 

At the next step, we examined the influence of chemotherapy and radiation therapy on the risk of AD death by comparison of AD mortality rates stratified by race and 5-year age intervals, specifically for the breast cancer (BC) patients, using the SEER 13 registry data. This registry was selected in order to limit a potential influence of the calendar period on the results, using the advantage of a narrower inclusion period of this registry (1992–2016), for which a higher number of records were available compared to the SEER 9 registry. 

During 1992 to 2016, SEER 13 recorded 120,881 white women diagnosed with breast cancer of invasive ductal carcinoma (IDC) type. The mean age at cancer diagnosis was 75.26 years. Chemotherapy was recorded as administered to 21,196 patients and 99,685 patents have a status recorded as “no/unknown”. Radiation therapy with beam radiation (or combinations of beam with other sources) was reportedly administered to 52,644 patients. “No/unknown” radiation treatment status was found for 63,343 patients, and 4894 patients had “other” status (as defined in the Materials and Methods section). During the follow-up time (mean = 6.73 years), 2713 patients died of Alzheimer’s disease at mean age 88.78 years. Additional cohort characteristics are presented in Table S4 (chemotherapy) and Table S5 (radiation therapy). 

Comparison of age-specific mortality rates indicates that chemotherapy significantly decreased the AD-specific mortality rate in breast cancer patients, when assessed for all follow-up times combined and within 119 months since cancer diagnosis (Table 3). This finding was consistent across four 5-year age groups encompassing the age period of 65 to 84 years, with overall crude and adjusted AD mortality rate ratios (MRRs) as follows: MRR = 0.4123 (CI95: 0.3521–0.4827) and MRR_MH_ = 0.5728 (CI95: 0.4884–0.6719), respectively. The Breslow-Day test for interaction over the age groups indicated no significant multiplicative interaction between age at cancer diagnosis and chemotherapy on the risk of AD death (*p* = 0.9765). However, a significant interaction between the effects of age at cancer diagnosis and chemotherapy status on AD mortality rate was found on an additive scale (Breslow-Day test for AD mortality rate differences *p* = 0.002533). Similarly, chemotherapy-treated groups displayed lower AD-specific mortality rates for follow-up times 120+ months since diagnosis (age groups 65–79 years), but the corresponding MRRs were not significantly different from 1. 

This comparison of crude mortality rates was performed for a single race across subgroups with very similar mean ages at cancer diagnosis, mean ages at AD death and calendar years of cancer diagnosis and the years of AD deaths (Table S4). As a result, the finding of reduced risk of AD death by chemotherapy is not likely affected by confounding. Furthermore, the strength of evidence is supported by a consistently protective effect of chemotherapy across several age groups.

BC patients treated with beam radiation displayed significantly lower age-specific AD mortality rates relative to the “no/unknown” treatment groups across five 5-year age groups (65–89 years) at all follow-up times combined (Table 4). Moreover, age-specific AD mortality rates were significantly lower in beam radiation-treated patients within 119 months since cancer diagnosis (in three age groups) and at 120+ months since cancer diagnosis (in one age group). For six age groups encompassing 65–94 years, the crude and age-adjusted AD mortality rate ratios were MRR = 0.602 (CI95: 0.556–0.652) and MRR_MH_ = 0.762 (CI95: 0.703–0.827), respectively. The interaction between age at diagnosis and radiotherapy status was not significant on a multiplicative scale (Breslow-Day test for MRR across age groups, *p*-value = 0.1703), but the interaction was significant on an additive scale (Breslow-Day test for AD mortality rate differences across age groups, *p*-value = 0.000618).

Groups with non-beam radiation therapy treatment (the status “other”) had too low absolute numbers of observed AD-deaths (O < 10) in most age intervals. Consequently, the corresponding mortality rates and MRRs were not determined for these groups. Only for the age groups 70–74 years, 75–79 years and 80–84 years, the absolute numbers of AD-associated deaths were 10 or more per group at all follow-up intervals combined. Corresponding MRRs suggested a decreased risk of AD death among the treated patients relative to the “no/unknown” treatment group; however, the result was significant only for the age group 75–79 years (MRR = 0.554; CI95: 0.292–0.593; *p* = 0.0338). 

Interestingly, breast cancer patients treated with chemotherapy and/or beam radiotherapy also displayed lower AD mortality rates than the reference populations (Tables S6 and S7). Patients diagnosed with cancer at age groups 70–74 years and 75–79 years, who were treated with chemotherapy, displayed lower overall risk of AD death than the corresponding reference populations, while patients with “no/unknown” chemotherapy status showed no overall reduction of the risk of AD death (Table S6). Patients treated with beam radiation had lower risk of AD death than reference populations at follow up times of 12 to 59 months (age groups 65–69 years, 70–74 years) and 60 to 119 months (age group 70–74 years). In patients diagnosed with cancer at 70–74 years of age, the risk of AD death was increased in the “no/unknown” chemotherapy group but not in the chemotherapy-treated group. The results for this age group imply a protective effect of beam radiation therapy for the time interval up to 120 months since cancer diagnosis, which is reversed at follow-up times starting 180 months since cancer diagnosis (Table S7). 

#### 2.4.2. Analysis of SEER 9 Registry Data

Additional analyses of the influence of chemotherapy and radiotherapy were performed on a larger cohort of breast cancer patients diagnosed over a longer time period in the SEER 9 registry. Between January 1975 and December 2016, SEER 9 registries recorded 405,799 patients diagnosed with invasive ductal carcinoma of the female breast as the only diagnosed cancer or the first diagnosed malignant primary cancer. From this group, we removed 928 patients diagnosed with benign tumors preceding breast cancer diagnosis, as well as 6174 patients with recorded person-time < 0.04 years. Among the remaining 398,677 patients, 2686 who died of any cause prior to the year of 1979 were removed from the cohort, because AD started to be recorded as a cause of death in 1979. The remaining cohort included 395,991 women diagnosed with breast cancer at median age of 60 years. During the follow-up time (with median at 7.25 years), 3894 of these patients (0.98%) died of AD (additional cohort characteristics are available in Table S8). The crude AD death rate during the follow-up time was 103.68 per 100,000 person-years. An additional 604 patients, who died of AD after being diagnosed with a second primary cancer, were not included among the AD cases, and their follow-up time was taken up to the time of diagnosis of second cancers. 

Considering limited follow-up times, the analysis was restricted to 337,267 women diagnosed with breast cancer at the age of 45 years or above, because they were more likely to have been followed-up to the age with more significant AD-specific mortality (characteristics of this cohort are presented in Table 5). Of these patients, 3876 (1.15% of the cohort) died due to AD over the follow-up time (with median equal to 7.13 years) at median age of 1065 months (88.8 years) with crude mortality rate of 126.22 per 100,000 person-years. 

The baseline cumulative hazard functions, estimated by the stratified Cox proportional hazards model for breast cancer patients, suggest non-proportional hazards over follow-up time among specific races (Figure S2). For instance, the point estimate of cumulative hazard ratios (cHRs) between white and black patients is 3.5 at 120 months and 1.9 at 240 months, which implies a changing influence of the race over the follow-up time. In an attempt to estimate relative hazards of AD death for different races, we used the Cox proportional hazards model stratified on the age at diagnosis (5 groups) with race as a single covariate (stratum and variable statuses are shown in Tables S9 and S10, respectively). The results indicate that the rate of AD death is significantly lower for API women (HR = 0.55; CI95: 0.46–0.65; *p* = 1.7 × 10^−11^) and black women (HR = 0.75; CI: 0.63–0.89; *p* = 7.5 × 10^−4^) relative to white women across the age groups. Similarly, the hazard ratio for AIAN women suggested lower risk relative to white women, but the difference was not statistically significant (HR = 0.70; CI95: 0.31–1.56). The omnibus test of model coefficients is significant (the deviance, –2 log-likelihood (–2LL) = 69,849.8; *p* = 2.4 × 10^−12^). The log(-log(survival)) against log time plots for 5 age groups do not show obvious violations of the proportional hazards assumption (Figures S3–S7); nevertheless, correlation of scaled Schoenfeld residuals versus log(time) suggests the non-proportionality for the black race even after the adjustment for age at cancer diagnosis (rho = 0.033; Chi-sq = 4.099; *p* = 0.0429; Table S11). Considering this observation, as well as high censoring rates and different censoring patterns among the race groups (Tables S9 and S10), our interpretation of these results is restricted to the reported detection of lower hazard of AD death for API and black female BC patients relative to white female BC patients, with a caveat of possibly biased estimates of hazard ratios. On the other hand, the plots of modeled survival curves imply a consistent pattern of AD hazard across races for all 5 age groups, at least for follow-up times beyond ~50 months (Figures S3–S7). Nevertheless, due to the identified non-proportionality in the effect of race on AD hazard rate over follow-up time, further analyses were limited to women of white races, which also represented a subgroup with the highest number of BC cases, highest number of AD deaths and longest average follow-up time (Table 5).

The Cox model for hazard of AD death using the age at diagnosis as a single variable (continuous) estimated an increase of the hazard of AD death in white women as ~18% per each additional year at cancer diagnosis (coefficient = 0.164; standard error SE = 0.002; *p* < 2 × 10^−16^). This model does not seem to invalidate the proportionality assumption (Supplementary Materials, Section S4.2A). Both chemotherapy and radiation therapy statuses indicated non-proportional hazards over follow-up time, when explored by using stratified Cox models (Figures S8–S11). Alternative approaches to Cox proportional modeling (Supplementary Materials, Section S4), including extensions of the Cox model to address the non-proportionality, did not remediate the issue of multiple non-proportional variables even when restricting the analysis to a single race. Moreover, this modeling strategy did not address the problem of competing risk events, such as the death due to other cancer and non-cancer causes, which substantially exceeded the risk of AD death in our cohort. 

**Figure 3:**
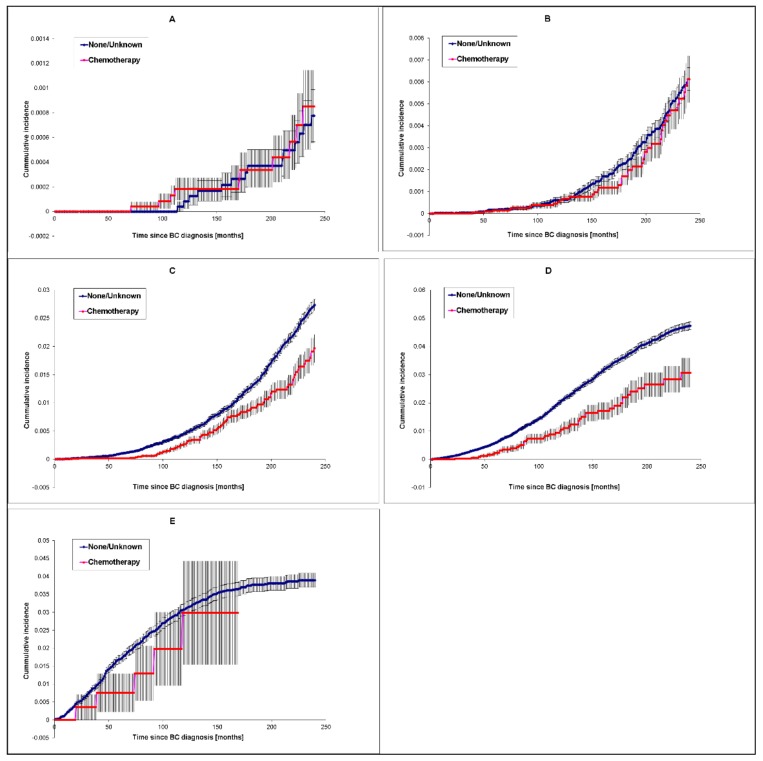
Effect of chemotherapy on cumulative incidence of AD death for white women diagnosed with breast cancer. Data for the age groups of 45–54 years (**A**), 55–64 years (**B**), (65–74 years (**C**), 75–84 years (**D**), and 85+ years (**E**) are shown. Error bars indicate standard errors (SEs).

For these reasons, we studied the influence of chemotherapy and radiotherapy on the risk of AD death using the analysis of competing risks. This analysis included women of white race at 5 separate age groups at cancer diagnosis (age group 1–5). Cumulative incidence (of mortality) plots illustrate the differences among age groups in the risk pattern over the time since cancer diagnosis. Chemotherapy-treated patients diagnosed at the age of 65–74 years (*p* = 6.55 × 10^−3^) and 75–84 years (*p* = 1.35 × 10^−4^), display a consistently lower risk of AD death over 240 months of follow-up than patients classified as having “no/unknown” chemotherapy status (Figure 3A–E). In contrast, the differences in cumulative incidence distributions were not significant for age groups 45–54 years (*p* = 0.2610), 55–64 years (*p* = 0.668) and 85+ years (*p* = 0.411). These differences imply that chemotherapy was protective at the ages when AD occurs more substantially, except for the 85+ years age group, where the result of the statistical test was affected by a small sample size. 

**Figure 4:**
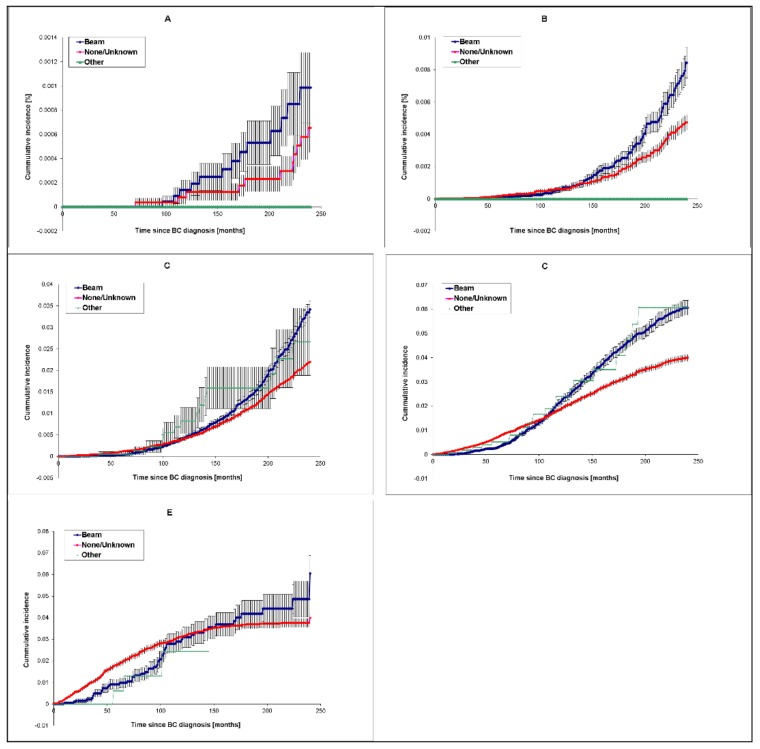
Effect of radiotherapy on cumulative incidence of AD death for white women diagnosed with BC. Data for the age groups of 45–54 years (**A**), 55–64 years (**B**), (65–74 years (**C**), 75–84 years (**D**), and 85+ years (**E**) are shown. For simplicity, only data for the beam radiotherapy are presented. Error bars indicate standard errors (SEs).

Differences in cumulative incidence curves among beam radiotherapy, other radiotherapy and no/unknown radiotherapy groups were significant for age groups 55–64 years (*p* = 9.04 × 10^−3^), 65–74 years (*p* = 2.61 × 10^−8^) and 75–84 years (*p* = 5.29 × 10^−7^). Similar to chemotherapy, protection from AD death by radiotherapy becomes more pronounced when radiotherapy is applied at more advanced ages, when AD occurs more frequently. However, in contrast to chemotherapy, the protective effect of beam radiotherapy is only seen at relatively early follow-up time intervals after cancer diagnosis (and presumably early after the treatment with beam radiation), while at longer follow-up time intervals, it is reversed to a deleterious effect. Cumulative AD incidence curves for beam radiotherapy-treated and untreated/unknown groups appear to cross during the follow-up time starting with age group 55–64 years, indicating the reversal of the effect with an increase in length of the follow-up time interval (Figure 4). This phenomenon is most prominent in the age group 75–84 years (Figure 4D), where beam radiotherapy appears to reduce the risk of AD death within the first 100 months since cancer diagnosis, but to increase risk at later follow-up time intervals. For instance, in this age group, the point estimate of cumulative AD mortality ratio (CMR) between beam radiation and “no/unknown” radiation groups changes, from 0.1 at 25 months to 0.5 at 70 months, and 1.5 at 200 months. A similar pattern is observed for other age groups, showing substantial risk reductions early after cancer diagnosis and a later increase in cumulative mortality ratio up to about 1.5-fold (Figure S12). The differences in cumulative mortality distributions among radiation therapy statuses over follow-up times were not significant for age groups 45–54 years (*p* = 0.618) and 85+ years (*p* = 0.657); nevertheless, the cumulative AD mortality in the 85+ years group was significantly lower in the beam radiation-treated subgroup within ~100 months since cancer diagnosis compared to the non-treated group. The highest protective effect of beam radiation in this age group was found at 35 months since BC diagnosis, when the cumulative AD mortality was 0.21% (CI95: 0.072%–0.52%) for beam-treated and 0.98% (CI95: 0.812%–1.177%) for the untreated group (absolute risk reduction 0.79% and corresponding relative risk RR = 0.22). Although the differences between beam radiation-treated and untreated subgroups in this age group are not statistically significant for later follow-up time intervals, the tendency of higher risk for the beam radiation-treated subgroup is still observed.

## 3. Discussion

### 3.1. Study Limitations

Our study addressed the links between two major health burdens faced by humankind by attempting to provide an etiological insight through the analysis of associations between cancer occurrence and AD mortality using data from population-based cancer registries from the Surveillance, Epidemiology and End Results (SEER) program. This study is subject to several potential limitations, which are discussed below to allow an objective assessment of our results in the context of methodological strengths and limitations. 

#### 3.1.1. Potential AD-Related Death Reporting Biases

First of all, cancer cases and AD death events included to our analysis were identified from the records in the National Cancer Institute (NCI) SEER Program cancer registries, SEER 9 and SEER 13. The SEER registry data have been previously used for evaluation of the risk of specific cancer [30] and non-cancer [31,32,33] mortalities among cancer patients in the United States and published in peer-reviewed journals. The SEER data are considered to be the gold standard for data quality amongst cancer registries in the US and globally [28]. Nonetheless, limitations that generally affect SEER observational studies also apply to our study. These include underreported and incomplete data on cancer therapy, variations in data reporting, selection bias and migration of patients from or to SEER registry regions [34]. 

Our study utilizes cause-specific death due to AD from the SEER variable “COD Recode 1969+” as an outcome of interest. This variable provides underlying causes of death recoded from the death certificates based on ICD 8–10. Available evidence supports underreporting of AD death on death certificates in the United States [4], which is likely caused by (i) reporting more general disease entities as a cause of death (e.g., dementia) instead of AD, (ii) misclassification of AD for other specific dementias or (iii) reporting more proximal causes of death, such as, for example, pneumonia, in cases where AD actually represents an underlying cause. As a result, the numbers of recorded AD deaths may underestimate the true numbers of AD deaths. Assuming that this underreporting applies comparably to our cancer cohorts and corresponding reference populations, our results based on SMR estimates are not expected to be substantially biased. In principle, we cannot rule out that to a certain degree, the underreporting of AD deaths could have disproportionately affected individuals with cancer history, whose death could have been more likely attributed to cancer even if their underlying cause of death was AD. However, if this bias would be present, we would be unlikely to find an increased risk of AD death in elderly cancer patients relative to the reference population, which is presented in Figure 1. In fact, in the group of elderly cancer patients, one can expect even more prominent bias towards overreporting cancer deaths, and underreporting other causes of death, including AD, and yet our results suggest an increased risk of AD death in this group of patients, which implies a low risk of the ascertainment bias. Another limitation related to the attribution of AD deaths stems from changed definition of AD death from ICD-9 (1979–1998) to ICD-10 (1999+), but this does not affect SMR estimates, because their calculation accommodates differences in years of diagnosis.

#### 3.1.2. AD Mortality versus AD Incidence

In this study, we employed AD mortality rates in cancer patients instead of AD incidence rates, which might better reflect the risk of AD occurrence in cancer patients. Mortality rates and incidence rates capture different aspects of the same dynamic process [35]. Therefore, differences in AD mortality rates between cancer and reference populations may partially or fully reflect differences in survival for AD in patients with and without a cancer diagnosis, which can be misinterpreted as differences in the risk of development of AD between the two groups. Nevertheless, a longitudinal study in a cohort of AD patients followed over two decades found no effect of baseline comorbidities (diabetes, hypertension, coronary disease, hyperlipidemia and cerebrovascular disease) on the survival of AD patients [36]. As a result, the clinical course of AD appears to be invariable against these and possibly other comorbidities, presumably including most cancers. Taken together, increased risk of AD death, found in our study for some cancer cohorts, is not likely related to decreased AD survival, but rather to increased risk of AD development. 

In contrast, however, the decreased risk of AD death found for some subgroups with baseline cancers could be explained by a better AD-specific survival. This is supported by a study that reported a slower rate of cognitive impairment in 75-year-old individuals who had been diagnosed with cancer within the past 10 years [37]. Since better cognitive ability and less impaired basic functional capacity are independent prognostic factors of longer survival [38], we cannot rule out that the decreased risk of AD death in cancer patients relative to individuals with no cancer history results, at least in part, from prolonged AD-specific survival of individuals with cancer history. Nevertheless, the consistency of our findings with incidence-based studies, such as, for example, the national veterans study [39], suggests that the possibly increased AD survival in cancer patients is not entirely responsible for the decreased AD mortality observed in our study.

#### 3.1.3. Limitations of the SMR Value Estimates

The associations between cancer and the risk of AD death was assessed using the standardized mortality ratio (SMR) values. SMR compares the observed number of AD deaths arising in the group of cancer patients with the number expected to occur on the basis of AD death in the reference population. The underlying assumption behind the use of SMR is that the rate of AD deaths in the cancer-free population can be approximated with the rate of AD deaths in the reference population. However, the reference populations would always include some proportion of cancer cases, and so the rate of AD death in reference populations entirely excluding cancer cases is not available. Consequently, we acknowledge this bias, whose extent depends on the SMR value and the prevalence of a condition in the general population. In BC, which is one of the most prevailing cancer types, with a frequency estimated as ~5% among women aged 45 years or over on January 2019 [40], the departure of the true relative risk from the observed (biased) SMR < 3.0 would be less than 10% [41]. For all cancers combined, complete prevalence is estimated to be < 1% in the age group of less than 50 years old, and ~12% in the age group of 50 years old or over [42]. Consequently, the calculated SMR indeed provides somewhat biased estimates of relative risk for all cancers combined, however it can depart by more than 10% from calculated SMR values only for patients aged ≥50 years and only for SMR values below 0.5 or above 1.5. Nevertheless, since this bias always tends to minimize or negate the observed effect sizes, the findings reporting increased risk or decreased risk relative to the reference population based on SMRs are not invalidated with respect to their direction and may only underestimate (but never overestimate) the magnitude of increased or decreased risk. 

#### 3.1.4. Treatment Underreporting 

Chemotherapy and radiotherapy treatment data in SEER registries were previously shown to underreport the administration of chemotherapy and radiotherapy. Sensitivities for chemotherapy and radiotherapy reporting were estimated as ~68% and ~80%, respectively (assessed for specific age group of patients diagnosed with any of seven selected cancer sites between 2000 and 2006) [43]. On the other hand, specificities for reporting exceeded 90% for both treatment types. As a result, patients recorded as treated were highly likely to have received the treatment, but some patients, who were not recorded as treated, could have in fact received the treatment, which was missed by the registry. For this reason, this group was labelled as “untreated/unknown”. We acknowledge that due to this incompleteness of data, our results of decreased risk of AD death in treated groups could have been biased towards the null hypothesis (that is, lack of effect). However, this does not invalidate our findings when differences are detected, but rather suggests that the actual effect of a treatment on the risk of AD-related death could have been even higher than the effect reported in our study. 

#### 3.1.5. Potential Heterogeneity of AD and Cancer Cases

One could suggest that aggregating all cases of AD death into a single outcome could conflict with well-established etiological and clinical heterogeneity of this disease. To account for this heterogeneity, some of our analyses separated substantially different early-onset AD (that includes most familial cases and all autosomal dominant AD cases) and late-onset AD (mostly sporadic) cases. While it has been argued that both early-onset [44] and late-onset AD [45] may still represent heterogeneous groups encompassing different disease etiologies and natural histories, the bulk of accumulated evidence and recent guidelines by the National Institutes of Health (NIH) and Alzheimer’s Association point to striking similarities in the underlying molecular foundations of all AD cases, as AD is always associated with the accumulation of amyloid β (Aβ) plaques and tau protein tangles [46]. While we acknowledge that these common key molecular events can be influenced by different genetic and/or environmental events, so that some etiological heterogeneity may have remained within our AD groups, these common features appear to be sufficient for studying an impact of cancer on AD in general.

The situation with cancer is more complicated, as cancer represents a very heterogeneous group of diseases. To the extent possible, we tried to accommodate this heterogeneity by separate analyses for cancers affecting specific sites. In addition, our analyses were performed separately for patients diagnosed with cancer at <45 years and those diagnosed at ≥45 years of age so that we could distinguish between the effects of etiologically different sporadic (mostly late onset) cancers and early onset cancers, which are more frequently associated with familial cancers and hereditary cancer syndromes [47].

#### 3.1.6. Multiple Comparisons

Multiple testing may identify a number of statistically significant associations by chance. In this study, we followed the approach taken by the National Cancer Institute (NCI) SEER program [48] and reported significant associations without multiple testing adjustments and argue for their interpretation in the light of (i) previous studies that identified inverse associations between Alzheimer’s disease and cancers, (ii) internal consistency of our findings across different cancer types and (iii) biological plausibility of our findings supported by mechanistic insight indicated in our discussion.

### 3.2. Interpretations of the Cancer-AD Association Data

For cancer patients diagnosed at ≥45 years, our calculated standardized mortality ratio (SMR) values generally support previously reported inverse associations between cancer and the risk of AD [49,50,51,52]. As indicated among the limitations of our study, decreased AD mortality rates in cancer patients are not necessarily reflective of decreased de novo development of AD (i.e., AD initiation), but they may reflect decreased progression of AD during its natural history, resulting in prolonged asymptomatic or symptomatic stages of the disease. 

In addition to previous reports, we have also uncovered a decreased risk of AD death for several specific solid tumors and hematolymphopoietic malignancies for white patients diagnosed with cancers at age ≥45 years, but not for the API patients. The results for black patients suggest a generally similar pattern as found for white patients (with the exception noted below), but the confidence is low due to lower accumulated person-years and the lack of statistical significance of SMR difference from 1 for most cancer types. Across several cancer types, our results for white patients are consistent with the results reported by a national veterans cohort study [39], with notable exceptions of melanoma and prostate cancer, for which the veterans study found increased risk of AD, while our results suggest decreased risk of AD death for melanoma and no difference in the risk of AD death for prostate cancer patients. However, our results for melanoma are supported by a large single-center US population study, which also found an inverse association between melanoma and the risk of AD [53]. The difference between the results of our study and those from the national veterans study for prostate cancer may stem from a possible difference between the prostate cancer cohorts in the use of androgen-deprivation therapy (ADT), which was found by some but not all investigators to increase the risk of AD [54]. Nevertheless, a recently reported Swedish study found an increased risk of dementia but not AD in prostate cancer patients treated with gonadotropin-releasing hormone agonists (HR = 1.15; CI95: 1.07–1.23) or orchiectomy (HR = 1.60; CI95: 1.32–1.93), and no increased risk for any type of dementia (of which, majority are usually due to AD) in men treated with oral antiandrogen or managed by watchful waiting [55], which is consistent with our results.

### 3.3. The Impact of Race on Cancer-AD Association

Most prominent differences in the risk of AD death between black cancer patients versus the reference population, and white cancer patients versus the reference population, were noted for uterine cervix cancers diagnosed at ≥45 years of age. Black patients with cervical cancers display a significantly increased risk of AD death for the follow-up intervals of 60–119 months and overall, while the risk for white patients was lower than in the reference population during the follow-up intervals of 60–119 months, and comparable to the reference population for all follow-up times combined (Table 2). The causes underlying these differences are not known. Other investigators have identified racial disparities in incidence and mortality of cervical cancer, which may be related to the differences in tumor histology [56,57], stage at diagnosis [58], medical care received by the patients [58,59] or human papillomavirus (HPV) genotypes and their coverage by available vaccines [60]. Groups of black and white patients with cervical cancers included in our cohort displayed significant differences among stages at cancer diagnosis, histological types and grades, but not in mean ages at cancer diagnosis or in proportions of patients who received chemotherapy, or radiotherapy that included beam radiation (Supplementary Materials, Section S2). Whether and how these clinical or biological differences project into the observed differences in the risk of AD death between black and white patients with cervical cancers remains to be elucidated. It should be noted that disparities between the white and African-American populations in the incidence and prevalence [61] and survival of AD [62] have been reported previously (also see below). Moreover, the study of a prospective cohort found an increased risk of AD development (detected as a decreased time to first AD diagnosis) among minority participants (mostly black) with cancer history, but a decreased risk in white participants with cancer history, which supports our findings for white patients and implies the existence of some differences between white and black cancer patients regarding the effect of cancer on the AD risk [51]. However, lack of information about cancer types and the small number of minority participants in this study limit more comprehensive interpretations of the observed racial differences.

The absence of a protective effect of cancer diagnosis in API patients, which was observed across several cancer sites and/or follow-up times, remains to be elucidated. We hypothesize that genetic differences may be, at least in part, responsible for the observed difference in the risk of AD death between white and API cancer patients. This is supported by previously reported differences among genes underlying AD risk in different races, which may also project to differences between risk of cancer development. For instance, a study in Han Chinese populations was able to validate only 4 of a total of 8 genes identified by genome-wide association studies (GWAS) as susceptibility genes in populations of European origin [63]. Likewise, effects of AD-associated genes on cancer could be different for the two populations. One example is the apolipoprotein E (APOE) gene, whose *ε4* allele causes from 4- to 15-fold (by different estimates) increase in the overall AD risk [64] and also increases the risk of BC in the Asian but not in the white population [65]. 

The lack of reduction of AD mortality in API cancer patients could also be interpreted in the context of pre-cancer differences in AD risk and differences in survival after AD diagnosis between the races. API patients have a reportedly lower incidence of dementias than any other race [66]. In addition, API patients displayed the longest survival after AD diagnosis and the lowest mortality rates compared to all other races across all age groups at AD diagnosis, while white patients tended to exhibit the poorest survival and highest mortality rates [67]. Notably, African Americans have higher incidence, prevalence [61] and reportedly tend to live longer following the diagnosis of AD in comparison with whites [62]. Consistent with these reports, our results suggest the highest risk of AD death in white BC patients and the lowest risk in API patients, and are also suggestive of the changing effect of race on the risk of AD death during the follow-up interval, as demonstrated by about an 1.8-fold decrease of risk for white race relative to black race between 120 and 240 months since BC diagnosis. 

### 3.4. Cancer and Early Onset AD

In contrast to older patients, the reduction of the risk of AD death was not observed for any race group of young patients who received cancer diagnosis at age <45 years. This could be interpreted in the context of the relatively short follow-up times, which did not allow follow-up of these patients to ages with more prominent occurrence of late-onset (sporadic) AD (LOAD). As a result, inferences about association between cancer in young patients and LOAD cannot be deduced from the SEER 9 data. On the other hand, inferences about the risk of early-onset AD (EOAD) were also limited for this group, due to the low occurrence of EOAD. While results suggest a significantly increased risk of AD-related death in black patients diagnosed with cancer at an early age, most likely due to EOAD, this finding is based on a relatively low number of EOAD deaths distributed across a wide range of different cancer types, and the small number of observed EOAD deaths implies its limited confidence, while the lack of significant association with specific cancers limits its interpretability. Another complication is the considerable genetic and phenotypic heterogeneity of EOAD. Even though, in contrast to LOAD, EOAD is almost an entirely genetic disease with a heritability of 92–100%, it is caused by mutations in a variety of genes, as reviewed in Reference [44], although essentially, all these mutations studied in detail to date directly or indirectly influence production and/or aggregation of Aβ.

One intriguing, although at this point speculative hypothesis, could be that the increased risk EOAD among black individuals diagnosed with cancer at <45 years of age could be associated with the *BRCA1* gene, whose pathogenic mutations are associated with BC and other cancers, and were shown to be more prevalent among black women diagnosed with BC at ≤50 years of age relative to the young white breast cancer patients [68]. Female BC patients were the most represented group among young black cancer patients. Depletion of the BRCA1 protein has been reported in the neuronal cultures exposed to Aβ oligomers, in transgenic mice expressing human Aβ precursor protein (APP) and in brains of AD patients [69], while silencing of neuronal BRCA1 expression in murine dentate gyrus resulted in neuronal and synaptic plasticity impairments as well as in deficits of learning and memory [69]. BRCA1 overexpression subsequent to ischemia/reperfusion injury is known to reduce neuronal oxidative damage, decrease neuronal apoptosis and attenuate neurological deficits, likely through activation of the NRF2-mediated antioxidative pathway [70]. It is therefore possible that the BRCA1 deficiency not only causes cancer, but also facilitates the progression of EOAD and leads to earlier EOAD death. However, further studies are needed to test this hypothesis. 

### 3.5. Impact of Age and Time Since Cancer Diagnosis on Cancer-AD Association

To our knowledge, our analysis has clearly demonstrated, for the first time, that the effect of cancer on the risk of AD death is modified by age and depends on the time since cancer diagnosis. Cancers diagnosed at 90 years of age do not seem to provide any protective effect from AD death at any follow-up time. In fact, the risk of AD death is increased in these patients relative to the reference population. One possible explanation of this result could be that a substantial proportion of these patients have already developed AD before cancer diagnosis, thus cancer may aggravate the clinical course of existing AD through interfering with the overall health and defense systems of a human organism. However, this explanation is inconsistent with the recently reported finding of a slower rate of cognitive decline in 75-year-old individuals, who developed new cancers or who were diagnosed with cancer within the previous decade [37]. Considering this finding, the interpretation that diagnosis of cancer at these advanced ages actually increases the risk of developing new AD remains plausible and warrants further investigation. The reversal of some effects with the increase of the post-cancer period (as in the case of radiation treatment, also see below) also adds to the notion that the possible impact of cancer or cancer-associated treatments on AD initiation should be considered.

The decreased risk of AD death determined for the groups with younger age at cancer diagnosis may reflect either protection against AD progression, or impact on AD initiation, or differential sensitivity of individuals with and without early asymptomatic AD to cancer, or all of these factors. As asymptomatic period of AD development may exceed 10 years, it is possible that some of the observed effects are indeed associated with the disease progression or differential sensitivity to cancer. Nevertheless, AD patients display decreased cancer mortality [25]; therefore, the inverse associations observed in our study are less likely attributable to increased sensitivity of asymptomatic AD patients to cancer. 

It should also be noted that the progression of AD might be impeded by cancer or cancer-related interventions only before AD reaches a certain stage of pre-symptomatic development, which would be in line with the “point of no return” concept previously suggested for some neurodegenerative diseases [71]. This adds complexity and limits mechanistic interpretation of our epidemiological study at the moment, pointing to the necessity of further studies in this direction.

Regarding the effect of age at cancer diagnosis, we estimated an 18% increase in risk of AD death with each additional year at cancer diagnosis in white women diagnosed with breast cancer. Our result is consistent with the reported doubling of age-specific incidence rates every 5 years over the age of 60 [6]. The complex relationship between the hazard of AD death and age at BC diagnosis, including non-proportionality identified in our study, is in line with the previously reported quadratic age effect [72].

### 3.6. Impacts of Anti-Cancer Therapies on AD Death

#### 3.6.1. Effects of Chemotherapy. 

Cytotoxic chemotherapy has been traditionally recognized as a cause of cognitive impairment in cancer survivors that affects working memory, executive functions and processing speed. In contrast to AD, chemotherapy-induced cognitive impairment (also known as “chemobrain”) is typically subtle with functioning preserved in the normal range and not affecting retrieval of remote memories [73]. As a result, chemotherapy-induced cognitive impairment (CICI) and AD represent substantially different disorders. Mechanistic differences of these two conditions have been comprehensively reviewed by Butterfield [74], who argued that although both these disorders are associated with free radicals, they substantially differ in the origin and molecular targets of oxidative stress and opportunities for suppressive pharmacological interventions. Consistent with differences between the two conditions was a case report of unexpected improvement of dementia in a patient treated for multiple myeloma with cytotoxic agents melphalan, carmustine, cyclophosphamide and vincristine, and glucocorticoid prednisone [75]. This report was published in the late 1990s, when mainstream research in the field focused on CICI, but its authors arrived at a visionary conclusion that their finding may offer new insight into the pathogenesis and management of AD. More recently, a national veterans study found decreased risk of AD in cancer survivors who received anti-cancer chemotherapy (HR = 0.55–0.80), regardless of cancer type [39], while other investigators reported increased risk of AD development in anthracycline-treated breast cancer patients [76]. 

Our results support the protective effect of cancer-directed chemotherapy against AD-related death in BC patients. This effect is significant in older women and remains protective over all follow-up times since cancer diagnosis. Interestingly, several anti-cancer agents are known to target microtubules trough inhibition of polymerization of tubulins (e.g., vinca alkaloids) or stabilization of microtubules (taxanes). This mode of action provides a plausible possible mechanistic link to their potential disease-modifying activity in AD, because of the recognized role of the microtubule-stabilizing protein tau in the pathogenesis of AD. Indeed, microtubule stabilizing agents were shown to suppress tau-mediated axonal dysfunction, neurotoxicity and cognitive deficits on in vivo models, which suggested the possible therapeutic potential of these anti-cancer agents in Alzheimer’s disease [77,78]. Pharmacological modulation of tau seems to be a promising treatment of AD also in the context of the findings from a cohort study, which reported lower amounts of paired helical filament tau neurofibrillary tangles among AD death cases with a history of cancer proximate to death compared to AD cases without cancer history, while both these two groups had comparable loads of amyloid-β plaques in brain tissues [52]. While our results do not link specific anti-cancer agents with a protective effect against AD death in cancer patients, we infer that these effects are related to some of the agents that were previously or more recently used for the treatment of breast cancers either as single agents, or in combinations. These agents include methotrexate, cyclophosphamide, 5-fluoruracil, doxorubicin, epirubicin, gemcitabine and taxanes. The extent to which these drugs penetrate the blood-brain barrier (BBB), and reach the brain tissue, warrants further investigation, and may be plausibly different for intact brain, brain with metastatic lesions of breast cancer or AD-affected brain. Answering this question would be necessary to develop an insight into the mechanisms of anti-AD activity of these anti-cancer agents. This will include elucidation of the place where these agents trigger their anti-AD initiating events, which may be within or outside the brain tissue. If these agents act in brain, their delivery through the BBB can be modulated [79], and so the efficacy of the treatment can be substantially improved. 

It should also be noted that there is emerging evidence connecting some forms of cancer to the accumulation of some proteins in an amyloid form (reviewed in Reference [80]), including a major tumor suppressor p53 [81,82]. Thus, it is possible that some anti-cancer therapies may also have a general anti-amyloid effect.

Consistent with Bradford-Hill’s criteria [83], further evidence for causation versus association of cancer-directed therapies with decreased risk of AD death would benefit from analysis of the effect of duration of therapies, with anticipation of lower risk of AD death in patients who received longer courses of these therapeutic interventions. This can be addressed by future studies, since data used in our studies do not allow for such a detailed analysis.

#### 3.6.2. Effects of Radiotherapy

Our study has uncovered the protective effect of beam radiation against AD death in women who received radiotherapy for BC. We specified that this protective effect operates only in older BC patients. Since 91% of radiotherapy-treated female breast cancer patients diagnosed at ≥65 years receive radiotherapy within the first two months from cancer diagnosis [84], our results suggest that the protective effect operates early after the radiotherapy is administered. With elapsing time, the protective effect of radiotherapy reverses to a deleterious effect, with an increased AD cumulative mortality ratio to about 1.5-fold relative to patients not treated with radiotherapy. To the best of our knowledge, such findings have not been reported previously. Although the data used in this study do not include details on radiation doses, fractionations or techniques, others have estimated the mean unintended dose to the brain ~0.2 Gy from the breast cancer radiotherapy (that included supraclavicular fields) using a 50 Gy tumor dose with beam energy of 6 MV photons (the dose to the spinal cord would reach 0.3–19 Gy) [85]. 

A study on the national cohort of veterans reported no protective effect of radiotherapy against development of AD [39]. Previously reported epidemiological studies that identified numerous risk factors for AD [86], including an extra copy of the chromosome with the APP (Aβ precursor)-coding gene in Down’s syndrome [87], as well as old age [6,12], obesity [6,13], type 2 diabetes [6,14], smoking [6,15,16] and electromagnetic fields [88], failed to provide strong evidence for any association between ionizing radiation and AD. A case-control study of female nuclear reactor workers based on a small number of cases reported an increased risk of death from dementia (senile or pre-senile) for women exposed to total lifetime radiation doses of 5–49.9 mSv relative to women exposed to 0–4.9 mSv (odds ratio (OR) = 2.09; CI95: 1.02–4.29) and a significant dose-response trend [88]. In contrast, the study of 2286 atomic bomb survivors who were exposed in Japan in 1945 to the dose of ≤4 Gy at the age of ≥13 years found no relationship between radiation exposure and the development of AD [89].

Ionizing radiation has been speculated as a possible risk factor for AD mostly based on (i) analogy with cognitive dysfunctions observed in cancer patients who received radiation therapy by cranial irradiation, (ii) cellular and molecular changes in animal and in vitro studies that implied endothelial damage, impaired neurogenesis, and (iii) plausible mechanistic links, such as shared molecular mechanisms between AD and some pathological conditions induced by ionizing radiation (e.g., the damage induced by reactive oxygen species, ROS) (reviewed in References [90,91,92]). Contrary to the expectations raised by these mechanistic inferences, evidence from the animal models did not support the etiological role of ionizing radiation in the AD. In fact, X-ray cranial irradiation was found to be protective in a study that employed a model based on the APP/PS1 double transgenic mice that express chimeric human/mouse amyloid precursor protein bearing the Swedish mutation (Mo/HuAPP695swe) and a mutant human presenilin 1 (PS1–dE9) in the CNS neurons. The APP/PS1 mice show detectable deposition of β-amyloid plaques at 3 months of age, with a progressively increasing number and spatial distribution of plaques and noticeable impairment of memory and cognitive performance starting at the age of 6 months. The nature of affected genes and the clinical course suggest consistency of this model with human EOAD [93]. In these mice, X-ray beam irradiation reduced the number and size of Aβ plaques and decreased cognitive impairment. These effects were more prominent at the irradiation schedule 2Gy × 5 than in 1Gy × 10 (hypofractionation), suggesting the potential importance of radiation schedule for the protective effect [94]. Interestingly, one study that employed the same animal model reported cognitive impairment and increased β-amyloid accumulation in brains of male mice exposed to 10 cGy and 100 cGy of ^56^Fe particle radiation [95]. However, this study employed unique high-charge high-energy particles which limits inferences to other types of ionizing radiation such as γ-radiation or X-ray. Interpretation of this study and its relevance to humans is limited by the fact that enhanced Aβ accumulation was only observed in male mice, while a cognitive deficit was found in both male and female mice, and it was not clear whether radiation effects are specific to mutations in particular AD-related genes. 

The growing body of evidence suggests considerable differences in biological effects between a low-dose ionizing radiation (LDIR) (<10 cG) and conventional doses used in the radiation therapy. Broad relevance of LDIR to various medical applications (e.g., radiation diagnostic procedures, unintended radiation from radiation therapy to organs outside the treatment field), and other human activities, such as nuclear energy production, sterilization by radiation or space travel warrants consideration of its potential role in the development of human disease. Concerns about a possible role of LDIR in the development of AD were raised by studies that reported considerably different gene expression profiles of brains between mice exposed to whole body LDIR of 10 cGy and mice exposed to a reference dose of 2 Gy [96]. Pathway enrichment analysis of gene expression data identified over-representation of downregulated genes in LDIR murine brains in six pathways associated with memory and learning, including the amyloid processing pathway, which is known to be downregulated in the AD [96]. However, this potential association between LDIR and AD has been challenged by an in vivo study that reported no detectable alterations in the expression of 84 AD-associated genes and no evidence for a late accumulation of Aβ or learning/memory alteration in C57BL/6J mice that received total body irradiation at 10 cGy [97].

To sum up, available evidence provides conflicting understandings of the influence of ionizing radiation on the development of AD. Our results bring additional complexity to this issue, but also an additional insight that can support further investigation. Interestingly, it was shown that ultraviolet (UV) light increase a frequency of the loss of the yeast endogenous heritable prion ([*PSI^+^*]) [98,99], that is controlled by an amyloid form of the Sup35 (eRF3) protein [100]. Proliferation of the Sup35 amyloid in yeast shows striking similarities to the proliferation of Aβ and tau amyloids in human brains. It has been suggested [101] that UV light (and possibly other DNA-damaging agents such as ionizing radiation) affect yeast prion via the induction of the chaperone proteins that are involved in cellular responses to both DNA damage and proteotoxic stresses, and play a crucial role in the control of amyloid fragmentation and proliferation [100,102]. We hypothesize that the anti-cancer radiotherapy may also act on AD-associated amyloids via altering the chaperone profiles, that may antagonize amyloid proliferation in a shorter run, but increase formation of new amyloids in a longer run.

As we currently understand, only a small portion of the ionizing radiation can reach the brain as an unintended target in breast cancer radiotherapy (except for the wide beam radiation therapy (WBRT) in case of brain metastases), and there may be substantial gains from optimizing treatment field, as well as radiation type, dose and fractionation.

Importantly, the effect of cancer chemotherapy and/or radiation therapy does not seem to be fully responsible for the decreased risk of AD in cancer patients, because the lower incidence of AD was also reported for non-melanoma skin cancers, which are rarely, if ever, treated with radiotherapy and/or cytotoxic cancer chemotherapy [53]. 

One of the possible factors to be considered while thinking about possible mechanistic explanations for the inverse association between female breast cancer and AD death is estrogenic exposure. There is considerable experimental, epidemiological and clinical support for estrogenic exposure as a risk factor for breast cancers (reviewed in Reference [103]). In contrast, the growing body of evidence suggests an increased risk of development or severity of AD due to reduced estrogenic exposure in perimenopause and menopause [104,105,106]. Our analysis demonstrates lower risk of AD death in younger women diagnosed with estrogen receptor (ER)-positive breast cancers in comparison with ER-negative cases (Supplementary Materials, Section S5). Since pre- and peri-menopausal women with ER-positive BC are treated with ovarian ablation, which induces premature menopause, these results support the hypothesis of the protective role of estrogenic exposure and the deleterious effect of menopause on AD development. Nevertheless, the difference between these groups has not reached statistical significance; therefore, this potential link needs to be addressed more pointedly by future investigation. 

Possibly, there could be multiple pathways that lead to inverse associations between AD-related death and cancer. In addition to the effects on the formation and/or propagation of amyloids produced by Aβ and tau, these links could be related to other previously suggested processes, such as inverse regulation of apoptosis, telomeres, glucose metabolism, or an inverse outcome of the same processes in neurons and other somatic tissues, such as genomic instability, inflammation and cell cycle re-entry (reviewed in References [20,25,45]). Overall, the mechanistic understanding of the relationship between cancer and AD requires further investigations with the use of model systems. 

## 4. Materials and Methods 

### 4.1. Analysis of the Risk of Death due to Alzheimer’s Disease in Cancer Patients Relative to the General Population

The risk of death due to AD in cancer patients relative to the general population was determined as standardized mortality ratios (SMRs) between the observed number of AD deaths in studied cohorts and the number of deaths that would be expected based on the age-, sex- and year-specific AD mortality rates in a standard population. 

SMRs were calculated using the NCI SEER*Stat software (version 8.3.6 Built Aug 6 2019) following a previously described method [107]. Patients diagnosed with any first primary cancer in SEER 9 registries between January 1975 and December 2016 were included to the cohort. These registries cover approximately 9.4% of the US population [108] and the database contains records on 5,160,473 tumors [109]. Only cases with malignant behavior (i.e., no in situ cancers without apparent stromal invasion) were included. Cases based only on Death Certificate of Autopsy were excluded. Observed number of deaths due to AD (O) were recorded in the cohort. The person-years at risk of death due to AD (PYR) were accumulated from cancer diagnosis to (i) date of death, (iii) date of diagnosis of a new malignant tumor, (iv) date of last known vital status or (v) end of the study, whichever occurred first. The expected number of deaths due to Alzheimer’s disease subsequent to cancer diagnosis (E) was calculated by multiplying PYR stratified by age (5-year groups), race (whites, blacks, other races) and year of diagnosis (5-year intervals) by strata-specific AD mortality rate in the general population. The expected (E) and observed (O) numbers of deaths due to AD for each stratum were summed up and the SMRs were calculated as observed (O)/expected (E). The 95% confidence intervals for SMR were determined using the exact method [110]. SMR values were considered statistically significant at *p* < 0.05 if their 95% confidence intervals did not overlap 1.0. The analysis was stratified on race (White, Black, American Indian/Alaska Native and Asian/Pacific Islander), age at cancer diagnosis (less than 45 years or 45 years of age or more), time passed since cancer diagnosis (0–11 months, 12–59 months, 60–120 months and 120+ months) and specific cancer sites. Cancer sites were used as defined in the “Site recode B ICD-O-3/WHO 2008” variable (NCI SEER*Stat software). Data for some cancer sites were combined to increase the number of cases via aggregation of biologically and clinically similar disease entities (e.g., the results for oral cavity cancers aggregate the results for cancers of the lip, tongue, salivary gland, floor of mouth, gum and other mouth and tonsil). Similarly, results for Hodgkin and non-Hodgkin lymphomas were aggregated to include their nodal and extranodal forms. The data were analyzed for individual cancer sites with at least 100,000 person-years of the follow-up time in at least one race group. As a result, some cancers that are uncommon and/or very aggressive, such as, for example, pharyngeal, pancreatic, liver, or gallbladder cancers, mesothelioma or sarcomas were not individually analyzed for SMRs, but they were analyzed aggregated with other data for analyses that included all cancer sites, all solid tumors or all lymphatic and hematopoietic diseases (for details see Table S1). The analyses were performed separately for patients diagnosed with cancer at <45 years of age or diagnosed at 45 or more years of age. The age of 45 was selected to separate etiologically different sporadic cancers from early onset cancers, which are more frequently associated with hereditary cancer syndromes and familial cancers [47]. Additional details (NCI SEER*Stat software settings) are available in Supplementary Materials (Section S1). 

### 4.2. Analysis of the Influence of Chemotherapy or Radiation Therapy on the Risk of AD Death in Breast Cancer Patients 

Women diagnosed with breast cancer represent an appropriate group for the analysis of the influence of demographic and clinical factors on the risk of AD death, because the high incidence of female breast cancer and the relatively long survival of these patients allow to create sizable retrospective cohorts of individuals followed-up for adequate time intervals, during which substantial numbers of AD deaths can occur. In addition, therapeutic approaches for breast cancers frequently include chemotherapy and/or radiotherapy, which allows for examination of their effects on the risk of AD death in these patients. All analyses in this study were limited to patients diagnosed with invasive ductal carcinoma of the breast of no special type (IDC; ICD-O-3 code:8500/3) which accounts for approximately 80% of all invasive breast cancers [9]. This selection was made to form a more homogeneous disease group by not including other less frequent and biologically more distant histological types. 

The effects of demographic and treatment variables on the hazard of the occurrence of AD death in female breast cancer patients were estimated using the Cox proportional hazards model implemented in R 3.5.1 [111] using the package “Survival”. Age at breast cancer diagnosis was used either as a continuous variable, or as an ordinal variable with age groups 1 (45–54 years), 2 (55–64 years), 3 (65–74 years), 4 (75–84 years) and 5 (85+ years). Calendar year of breast cancer diagnosis was used as a continuous variable or an ordinal variable with four levels: 1 (1975–1984), 2 (1985–1994), 3 (1995–2004) and 4 (2005–2016). Race was used as a categorical variable and coded as described in previous section. SEER variable “Chemotherapy recode” was used with original levels “Yes” and “No/Unknown”. Variable “Radiation recode” was re-coded by grouping of categories as follows: Beam (Beam radiation + Combination of beam with implants or isotopes), None/Unknown (None/Unknown + Refused (1988+)), Other (Radioactive implants (includes brachytherapy) + Radioisotopes (1988+) + Radiation, NOS (method or source not specified) + Other than beam radiation (1973–1987) + Recommended, unknown if administered). This grouping was performed to estimate the influence of beam radiation on the risk of AD death relative to the “none/unknown” group that is most representative to patients not treated with beam radiation or any radiation therapy. The proportionality assumption was tested using Pearson product-moment correlation of scaled Schoenfeld residuals versus log(time) for each variable, and *p*-values < 0.10 were considered as indicative of violation of the proportionality assumption. 

Additionally, cumulative incidence of AD death (cumulative AD mortality) was estimated taking into account competing risk of death from all other causes using the “Cmprsk” package in R [112], and the significance of the difference among cumulative incidence functions for different chemotherapy or radiotherapy statuses was tested using Gray’s test [113]. 

The analyses were performed on the cohort of patients from SEER 9 registries who were diagnosed with primary female breast cancers of IDC histological type as first malignant tumors. SEER 9 data were collected between January 1975 and December 2016. Cases based on Death Certificate or Autopsy only were excluded. Information on the race (white, black, Asian/Pacific Islander and American Indian/Alaska Native), age at diagnosis, cause of death, chemotherapy status and radiation therapy status were collected. Time-on-study was determined for each patient as the time from breast cancer diagnosis to (i) date of death due to AD, (ii) date of death due to any other cause, (iii) date of diagnosis of any new primary tumor, (iv) last known vital status, or (v) end of the study, whichever occurred first. Time-on-study was used as time scale and the death due to Alzheimer’s disease was considered as an event of interest, while all other outcomes were censored (Cox model). In the competing risk model, the events of interest were (i) death due to AD and (ii) death due to all other causes, while all other outcomes were censored. Since AD was recorded as a cause of death only after the start of the International Classification of Diseases, Ninth Revision (ICD-9) in 1979, the patients with attained years prior 1979 were removed from the cohort. 

Further, the analysis of the effect of chemotherapy or radiation therapy based on the comparison of age-specific AD mortality rates was limited to white women from the SEER 13 registry diagnosed with primary breast cancer of IDC histological type as the first malignant tumor between January 1992 and December 2016. Cases based only on Death Certificate of Autopsy were excluded. Numbers of AD deaths and person-years were determined for seven 5-year age groups covering the interval between 65 and 99 years of age, separately for each chemotherapy or radiation therapy status, and for different time intervals since breast cancer diagnosis (0–119 months, 120+ months and all follow-up time intervals combined). The effect of chemotherapy or radiation therapy was determined for each age group and each follow-up time interval via comparison of age-specific Alzheimer’s disease mortality rates between subgroups that differed in these treatments. Differences between mortality rates were tested using the “Test based Method”-derived *p*-value and mortality rate ratios (MRRs) with 95% confidence intervals were calculated using the exact Poisson method [114] implemented in MedCalc Statistical Software version 17.9.7 (MedCalc Software, Ostend, Belgium). The Mantel–Haenszel method was used to adjust rate ratios across age strata if the Breslow-Day test for interaction of rate ratio over strata was not statistically significant (*p* > 0.05 determined by OpenEpi tool [115]). Subgroups with a single race, narrow age intervals and relatively narrow range of calendar years at cancer diagnosis were chosen to allow comparison of non-standardized mortality rates among subgroups of breast cancer patients that would be homogeneous by respective parameters and have sufficient numbers of AD deaths for all subgroups differing by chemotherapy or radiation therapy statuses. 

## 5. Conclusions

Our analysis of the risk of AD death in cancer patients relative to reference populations, stratified by race, cancer sites, age at diagnosis and time since cancer diagnosis, suggests a decreased risk of AD death in white patients diagnosed with different solid tumors or leukemias at the age of ≥45 years. This agrees with previous studies which reported inverse associations between cancer and the risk of AD [53,54,55,56]. However, our work identified specific solid tumors and hematolymphopoietic malignancies associated with a decreased risk of AD death in white patients and demonstrated modification of this protective effect by both age at diagnosis and time since cancer diagnosis. Notably, the protective effect of cancer against AD death was not detected in Asian/Pacific Islander patients, while in black patients, diagnosis of cancer at <45 years of age was associated with an increased risk of AD death, which was likely attributable to early-onset AD. Based on these results, and previous mechanistic and epidemiological studies [72,73,74], we hypothesized that the positive association between cancers in young black patients and early-onset AD death could be, at least in part, explained by the BRCA1 deficiency in these individuals. This hypothesis warrants further studies to confirm a possible etiological role of BRCA1 in the EOAD and develop targeted therapeutic or preventive interventions. The inverse associations between specific cancers and the risk of AD death, identified by our study, open new avenues for identification of shared risk factors and molecular mechanisms involved in the development of cancer and AD, the former being typically linked to DNA changes, and the latter being the most widespread disease associated with protein misfolding.

Importantly, our results showed the protective effect of chemotherapy against AD death in white women diagnosed with breast cancer at ≥65 years of age, and a more complex pattern of the effect of radiotherapy, exhibiting a decrease in the risk of AD-associated death at early time periods after treatment, followed by an increase in the risk of AD death at later times after treatment. These findings are plausible in the light of other epidemiological [45] or animal and mechanistic studies [80,81,96], and imply the possibility of future development of these approaches for the prevention or treatment of AD.

## Figures and Tables

**Table 1:** Cohort characteristics (all cancer sites aggregated).

Characteristics	White	Black	AIAN ^†^	API ^‡^	All Races
Number of cancer cases Age: 0–44 years Age: 45+ years	347,583 2,902,524	47,745 325588	3,970 17,361	33,787 212,982	433,085 3,458,455
Accumulated persons-years at risk Age: 0–44 years Age: 45+ years	4,233,862.49 17,226,235.60	419,384.02 1,547,446.29	38,158.83 81,733.97	324,710.40 1,116,766.56	5,016,115.73 20,022,182.42
Mean follow-up time (years) Age: 0–44 years Age: 45+ years	12.18 5.93	8.78 4.75	9.61 4.71	9.61 5.48	11.58 5.79
Number of deaths due to AD Age: 0–44 years Age: 45+ years	76 21,098	10 1,086	0 40	1 855	87 23079
Mean age at cancer diagnosis (years)	64.4	61.3	59.0	62.8	64.0
Mean age at AD diagnosis (years)	87.4	86.2	85.3	88.9	87.4

**^†^** AIAN: American Indian/Alaska Native, ^‡^ API: Asian/Pacific Islander, AD: Alzheimer’s disease.

**Table 2:** Analysis of the risk of death due to Alzheimer’s disease (AD) in cancer patients relative to the general population.

Tumor Site	Time	White			Black			American Indian/ Alaska Native			Asian or Pacific Islander		
		O	SMR	N	O	SMR	N	O	SMR	N	O	SMR	N
All Sites	0–11	831	0.58 *	2,902,524	90	1.07	325,588	0	0	17,361	55	1.12	212,982
	12–59	2961	0.66 *	2,017,523	149	0.61 *	211,235	5	0.72	11,068	110	0.77 *	144,601
	60–119	4781	0.96 *	1,163,005	246	0.91	107,434	11	1.6	5616	187	1.15	78,838
	120+	12,525	1.17 *	652,960	601	1.17 *	53,905	24	1.89 *	2840	503	1.47 *	42,731
	Total	21,098	0.97 *	2,902,524	1086	0.98	325,588	40	1.38	17,361	855	1.23 *	212,982
All Solid Tumors	0–11	677	0.54 *	2,591,705	75	0.99	294,314	0	0	15,459	48	1.09	193,045
	12–59	2671	0.66 *	1,836,126	140	0.62 *	194,165	5	0.79	10,075	104	0.79 *	133,998
	60–119	4477	0.97 *	1,072,839	236	0.92	100,169	11	1.69	5183	180	1.18 *	73,929
	120+	12,043	1.17 *	611,020	586	1.18 *	50,975	24	1.96 *	2670	478	1.45 *	40,480
	Total	19,868	0.99 *	2,591,705	1037	0.99	294,314	40	1.46 *	15,459	810	1.23 *	193,045
All Lymphatic and Hematopoietic Diseases	0–11	78	0.55 *	235,508	8	1.09	22,966	0	0	1341	2	0.5	15,144
	12–59	226	0.57 *	158,588	7	0.38 *	15,140	0	0	864	5	0.5	9282
	60–119	246	0.75 *	80,438	9	0.73	6519	0	0	380	7	0.85	4369
	120+	410	1	37,054	12	0.81	2557	0	0	145	21	1.90 *	1966
	Total	960	0.75 *	235,508	36	0.68 *	22,966	0	0	1341	35	1.05	15,144
Oral cavity	0–11	22	0.83	62,222	3	3.89	5925	0	0	326	0	0	3168
	12–59	54	0.74 *	48,305	1	0.5	3965	0	0	249	1	0.47	2379
	60–119	63	0.91	25,637	3	1.55	1624	2	17.06 *	109	3	1.59	1229
	120+	180	1.25 *	13,388	4	0.89	771	0	0	51	5	1.22	654
	Total	319	1.02	62,222	11	1.2	5925	2	3.94	326	9	1	3168
Stomach	0–11	7	0.33 *	49,259	1	0.38	8261	0	0	590	4	1.58	9623
	12–59	28	0.73	20,760	2	0.46	3524	0	0	209	3	0.59	4825
	60–119	32	0.97	6536	4	1.13	1152	0	0	63	9	1.84	1898
	120+	66	1.31 *	3107	9	1.72	522	0	0	32	13	1.46	998
	Total	133	0.93	49,259	16	1.02	8261	0	0	590	29	1.35	9623
Colon excluding Rectum	0–11	85	0.50 *	238,546	8	0.77	27,506	0	0	1286	3	0.49	19,030
	12—59	322	0.58 *	167,866	17	0.57 *	18,467	1	1.26	878	15	0.74	14,310
	60–119	612	1.03	92,691	36	1.12	9012	0	0	411	30	1.31	8039
	120+	1416	1.24 *	52,188	80	1.17	4863	4	3.29	197	81	1.71 *	4503
	Total	2435	0.99	238,546	141	1	27,506	5	1.69	1286	129	1.33 *	19,030
Rectum and Rectosigmoid Junction	0–11	20	0.43 *	98,430	3	1.27	9106	0	0	650	1	0.51	9946
	12–59	86	0.59 *	75,021	4	0.61	6615	0	0	480	10	1.62	7903
	60–119	157	0.97	40,212	7	1.02	3181	0	0	219	7	0.99	4224
	120+	518	1.23 *	22,940	28	1.75 *	1593	1	1.87	106	29	1.48	2325
	Total	781	1.01	98,430	42	1.32	9106	1	1	650	47	1.35	9946
Larynx	0–11	4	0.52	27,814	0	0	4384	0	0	125	0	0	1159
	12–59	17	0.65	22,479	2	1.16	3292	0	0	94	0	0	936
	60–119	24	0.86	12,778	3	1.64	1535	0	0	45	0	0	533
	120+	60	0.86	7090	6	1.32	762	0	0	19	3	1.77	289
	Total	105	0.80 *	27,814	11	1.27	4384	0	0	125	3	0.88	1159
Lung and Bronchus	0–11	111	0.78 *	400,070	14	1.53	50,838	0	0	1729	11	1.8	28,702
	12–59	114	0.56 *	152,240	9	0.76	18,785	0	0	635	4	0.47	11,950
	60–119	118	0.89	39,797	6	0.91	4117	3	22.59 *	147	5	1.03	2887
	120+	210	1.19 *	16,810	13	1.35	1600	0	0	51	5	0.72	1167
	Total	553	0.84 *	400,070	42	1.13	50,838	3	3.44	1729	25	0.94	28,702
Melanoma of the Skin	0–11	34	0.55 *	104,070	1	3.59	547	0	0	223	0	0	823
	12–59	178	0.9	89,593	0	0	432	0	0	185	1	1.1	674
	60–119	168	0.89	56,914	2	3.43	226	0	0	110	1	1.45	356
	120+	434	0.99	33,396	2	1.37	121	0	0	65	1	0.93	196
	Total	814	0.92 *	104,070	5	1.67	547	0	0	223	3	1.01	823
Female Breast	0–11	93	0.39 *	423,475	8	0.59	41,945	0	0	2413	1	0.15 *	33,017
	12–59	661	0.69 *	375,853	35	0.72	35,704	1	0.79	2111	18	0.69	29,167
	60–119	1155	0.98	256,413	36	0.69 *	20,683	1	0.66	1277	38	1.14	19,199
	120+	3466	1.18 *	157,281	150	1.25 *	11,184	7	2.28	713	122	1.29 *	11,428
	Total	5375	1.01	423,475	229	0.98	41,945	9	1.45	2413	179	1.12	33,017
Cervix Uteri	0–11	1	0.25	18,443	1	1.37	4096	0	0	284	0	0	2470
	12–59	7	0.62	14,118	2	1	3005	0	0	210	0	0	2008
	60–119	7	0.48 *	8452	8	3.13 *	1659	0	0	112	2	1.79	1261
	120+	87	1.09	5754	14	1.31	1062	0	0	78	10	1.46	898
	Total	102	0.93	18,443	25	1.56 *	4096	0	0	284	12	1.31	2470
Corpus Uteri	0–11	18	0.43 *	102,895	1	0.41	8278	0	0	595	0	0	7447
	12–59	70	0.44 *	89,195	8	1.21	6082	0	0	494	1	0.3	6273
	60–119	193	0.85 *	63,065	7	0.94	3028	0	0	295	2	0.45	4009
	120+	1296	1.17 *	43,117	19	0.91	1627	2	2.13	156	29	1.33	2488
	Total	1577	1.02	102,895	35	0.94	8278	2	1.34	595	32	1.05	7447
Ovary	0–11	16	0.84	49,631	0	0	3860	0	0	393	0	0	3296
	12–59	14	0.34 *	32,229	1	0.52	2145	0	0	264	0	0	2286
	60–119	25	0.7	13,185	2	1.26	769	0	0	112	1	1.03	1034
	120+	130	1	7228	6	1.52	369	1	4.17	57	4	0.93	575
	Total	185	0.82 *	49,631	9	1.06	3860	1	2.55	393	5	0.75	3296
Prostate	0–11	98	0.46 *	468,304	13	0.7	73,082	0	0	2334	8	1.03	28,745
	12–59	668	0.71 *	423,333	44	0.54 *	64,547	1	0.57	2037	29	0.83	25,887
	60–119	1306	0.99	291,592	103	0.9	41,597	5	2.41	1286	56	1.11	17,479
	120+	2842	1.19 *	160,290	212	1.12	21,057	4	1.35	638	114	1.49 *	9031
	Total	4914	1.01	468,304	372	0.92	73,082	10	1.38	2334	207	1.22 *	28,745
Urinary Bladder	0–11	64	0.59 *	143,356	5	1.36	7199	0	0	373	6	2.19	5977
	12–59	225	0.66 *	113,034	7	0.72	4875	2	10.85 *	257	8	1.01	4656
	60–119	321	0.96	68,104	7	0.84	2489	0	0	148	13	1.68	2672
	120+	697	1.16 *	37,795	11	0.9	1239	1	3.08	82	19	1.47	1422
	Total	1307	0.95 *	143,356	30	0.89	7199	3	4	373	46	1.47 *	5977
Kidney	0–11	29	1.04	66,840	3	1.61	8035	0	0	961	0	0	4314
	12–59	51	0.57 *	46,993	1	0.17 *	5486	0	0	667	4	1.28	3177
	60–119	83	0.91	26,341	6	1	2862	0	0	340	4	1.32	1685
	120+	180	1.19 *	13,376	9	1.03	1265	1	1.77	165	7	1.99	735
	Total	343	0.95	66,840	19	0.85	8035	1	0.68	961	15	1.42	4314
Thyroid	0–11	5	0.6	35,504	0	0	2865	0	0	310	0	0	4663
	12–59	16	0.48 *	30,928	1	0.55	2420	0	0	263	0	0	4014
	60–119	31	0.75	21,128	1	0.54	1507	0	0	158	3	1.33	2738
	120+	118	0.87	12,002	3	0.56	772	0	0	98	7	0.82	1570
	Total	170	0.78 *	35,504	5	0.53	2865	0	0	310	10	0.77	4663
Non-Hodgkin Lymphoma	0–11	31	0.44 *	112,554	4	1.84	8008	0	0	609	2	0.86	8242
	12–59	114	0.55 *	79,707	2	0.33	5366	0	0	396	5	0.75	5359
	60–119	146	0.75 *	44,300	4	0.74	2774	0	0	215	4	0.65	2881
	120+	268	1.04	21,932	7	0.79	1322	0	0	79	18	2.11 *	1386
	Total	559	0.77 *	112,554	17	0.76	8008	0	0	609	29	1.23	8242
Myeloma	0–11	15	0.67	36,877	2	0.66	8263	0	0	355	0	0	2665
	12–59	27	0.54 *	24,956	2	0.29	5711	0	0	241	0	0	1834
	60–119	17	0.71	8298	3	0.91	1929	0	0	67	3	3.84	603
	120+	17	1.25	2258	3	1.2	531	0	0	22	1	1.44	164
	Total	76	0.69 *	36,877	10	0.64	8263	0	0	355	4	1.03	2665
Chronic Lymphocytic Leukemia	0–11	18	0.58 *	36,054	0	0	2452	0	0	112	0	0	752
	12–59	69	0.68 *	30,396	2	0.49	1987	0	0	84	0	0	612
	60–119	64	0.75 *	17,676	1	0.36	1001	0	0	43	0	0	334
	120+	94	0.93	7951	1	0.46	346	0	0	19	2	2.1	141
	Total	245	0.77 *	36,054	4	0.39 *	2452	0	0	112	2	0.64	752

Time—follow-up time after cancer diagnosis. SMR—standardized mortality rates; *N*—number of cancer patients included. * *p* < 0.05 (not adjusted for multiple tests). Color coding: green—decreased risk of AD death relative to the general population; orange—increased risk of AD death relative to the general population.

**Table 3:** Crude Alzheimer’s disease mortality rates and mortality rate ratios (MRR) for breast cancer patients from SEER (Surveillance, Epidemiology, and End Results) 13 for different chemotherapy statuses. Mortality rates and MRRs were not calculated for subgroups with O < 10.

Age at Diagnosis (Years)	Time since BC Diagnosis (Months)	Chemotherapy Status								Crude MRR	CI95 ^c^	*p*-Value ^d^
		No/Unknown				Yes						
		O ^a^	Persons	Person-Years	Crude AD Mortality Rate ^b^	O ^a^	Persons	Person-Years	Crude AD Mortality Rate ^b^			
65–69	0–119	57	24,703	154,178.79	36.97	10	10,286	55,654.87	17.97	0.486	0.221–0.960	0.0315
	120+	205	8748	50,105.53	409.14	37	2541	11,404.05	324.45	0.793	0.543–1.130	0.1931
	Total	262	24,703	204,284.32	128.25	47	10,286	67,058.92	70.09	0.547	0.392–0.748	0.0001
70–74	0–119	127	23,885	147,381.40	86.17	14	6182	31,469.60	44.49	0.516	0.274–0.899	0.0168
	120+	387	8111	40,853.18	947.29	43	1310	5474.60	785.45	0.829	0.590–1.138	0.243
	Total	514	23,885	188,234.58	273.06	57	6182	36,944.21	154.29	0.565	0.422–0.744	<0.0001
75–79	0–119	296	21,724	127,501.65	232.15	19	3191	15,014.76	126.54	0.545	0.324–0.867	0.0092
	120+	339	6007	24,295.26	1395.33	23	549	1898.00	1211.80	0.869	0.543–1.325	0.512
	Total	635	21,724	151,796.92	418.32	42	3191	16,912.76	248.33	0.594	0.424–0.812	0.0009
80–84	0–119	420	16,205	82,793.72	507.28	13	1179	4695.96	276.83	0.546	0.288–0.943	0.029
	120+	213	2796	8314.72	2561.72	8	118	304.28	N/A	N/A	N/A	N/A
	Total	633	16,205	91,108.44	694.78	21	1179	5000.25	419.98	0.605	0.372–0.932	0.0218
85–89	0–119	308	9267	38,241.96	805.4	5	293	843.2	N/A	N/A	N/A	N/A
	120+	54	786	1740.11	3103.25	0	7	12.25	N/A	N/A	N/A	N/A
	Total	362	9267	39,982.07	905.41	5	293	855.45	N/A	N/A	N/A	N/A
90–94	0–119	104	3261	9809.39	1060.21	0	57	124.03	N/A	N/A	N/A	N/A
	120+	6	67	97.48	N/A	0	0	0	N/A	N/A	N/A	N/A
	Total	110	3261	9906.86	1110.34	0	57	124.03	N/A	N/A	N/A	N/A
95–99	0–119	25	640	1513.91	1651.35	0	8	25.25	N/A	N/A	N/A	N/A
	120+	0	3	1.25	N/A	0	0	0	N/A	N/A	N/A	N/A
	Total	25	640	1515.16	1649.99	0	8	25.25	N/A	N/A	N/A	N/A

^a^ Number of deaths due to Alzheimer’s disease. ^b^ Rate per 100,000 person-years. ^c^ 95% Confidence interval. ^d^ From a test-based method.

**Table 4:** Crude Alzheimer’s disease mortality rates and mortality rate ratios (MRR) for breast cancer patients from SEER 13 for different radiation therapy statuses. Mortality rates and MRRs were not calculated for subgroups with O < 10.

Age (Years)	Time since BC Diagnosis (Months)	Radiation Status								Comparison Beam: No/Unknown		
		No/Unknown				Beam				MRR	CI95 ^c^	*p*-Value ^d^
		O ^a^	Persons	Person-Years	Crude AD Mortality Rate ^b^	O	Persons	Person-Years	Crude AD Mortality Rate			
65–69	0–119	34	14,420	85,637.71	39.7	32	18,787	115,147.04	27.79	0.7	0.418–1.170	0.1454
	120+	126	4742	27,631.06	456.01	111	6244	32,758.58	338.84	0.743	0.571–0.967	0.022
	Total	160	14,420	113,268.77	141.26	143	18,787	147,905.62	96.68	0.684	0.542–0.863	0.0009
70–74	0–119	82	13,900	80,724.34	101.58	52	14,888	91,811.65	56.64	0.558	0.386–0.799	0.0008
	120+	218	4363	22,233.44	980.51	207	4866	23,350.98	886.47	0.904	0.744–1.099	0.2987
	Total	300	13,900	102,957.78	291.38	259	14,888	115,162.63	224.9	0.772	0.651–0.915	0.0022
75–79	0–119	180	13,140	71,581.47	251.46	126	10,769	65,982.38	190.96	0.759	0.6	0.959
	120+	190	3220	12,926.41	1469.86	168	3190	12,860.67	1306.31	0.889	0.718–1.100	0.2651
	Total	370	13,140	84,507.88	437.83	294	10,769	78,843.06	372.89	0.852	0.728–0.995	0.0397
80–84	0–119	300	10,963	51,835.34	578.76	123	5897	33,193.43	370.56	0.64	0.515–0.792	<0.0001
	120+	121	1635	4902.67	2468.04	94	1231	3615.37	2600.01	1.054	0.796–1.391	0.7048
	Total	421	10,963	56,738.00	742.01	217	5897	36,808.80	589.53	0.795	0.671–0.938	0.0058
85–89	0–119	265	7358	28,581.32	927.18	43	1948	9505.77	452.36	0.488	0.345–0.675	<0.0001
	120+	39	542	1130.28	3450.47	15	240	603.95	2483.65	0.72	0.369–1.336	0.277
	Total	304	7358	29,711.60	1023.17	58	1948	10,109.72	573.71	0.561	0.416–0.745	<0.0001
90–94	0–119	93	2943	8597.44	1081.72	10	329	1177.30	849.4	0.785	0.409–1.508	0.467
	120+	6	55	81.29	N/A	0	12	16.19	N/A	N/A	N/A	N/A
	Total	99	2943	8678.73	1140.72	10	329	1193.49	837.88	0.735	0.383–1.407	0.3505
95–99	0–119	24	619	1481.60	1619.87	1	26	52.9	N/A	N/A	N/A	N/A
	120+	0	3	1.25	N/A	0	0	0	N/A	N/A	N/A	N/A
	Total	24	619	1482.85	1618.5	1	26	52.9	N/A	N/A	N/A	N/A

^a^ Number of deaths due to Alzheimer’s disease. ^b^ Rate per 100,000 person-years. ^c^ 95% Confidence interval. ^d^ From a test-based method.

**Table 5:** Cohort characteristics for 337,267 breast cancer cases from the SEER 9 registry included into analysis of the effects of chemotherapy, radiotherapy and demographic/clinical factors on the risk of AD death.

Characteristics		AIAN ^†^	API ^‡^	Black	White	ALL
Number of BC patients		1726	24,993	28,456	282,092	337,267
Number of primary cancers per patient *	1	1531	21,660	24,667	237,452	285,310
	2–14	195	3333	3789	44,640	51,957
Chemotherapy	No	1011	15,882	16,497	202,063	235,453
	Yes	715	9,111	11,959	80,029	101,814
Radiotherapy	Beam	783	12,799	12,918	121,878	148,378
	No	867	11,695	14,246	151,129	177,937
	Other	76	499	1292	9085	10,952
AD deaths	No	1720	24,865	28,317	278,489	333,391
	Yes	6	128	139	3603	3876
Year of birth: Median (range)		1945 (1887–1971)	1942 (1883–1971)	1941 (1882–1971)	1933 (1876–1971)	1,935 (1876–1971)
Year of diagnosis: Median (range)		2005 (1975–2016)	2005 (1975–2016)	2003 (1975–2016)	1999 (1975–2016)	1999 (1975–2016)
Person-years: Median (range)		5.63 (0.08–37.42)	6.58 (0.08–41.47)	5.08 (0.08–41.88)	7.25 (0.08–41.88)	7.00 (0.08–41.88)
Age at diagnosis (years) Median (range)		58 (45–103)	60 (45–104)	60 (45–107)	63 (45–107)	63 (45–107)
Age at AD death (months) Median (range)		1008 (846–1113)	1082 (786–1245)	1055 (724–1264)	1065 (694–1274)	1065 (694–1274)
Time between BC and AD death (months): median (range)		199 (23–318)	175 (16–448)	176 (3–459)	160 (2–497)	161 (2–497)

^†^ American Indian/Alaska Native. ^‡^ Asian/Pacific Islander. ^*^ Includes in situ and malignant tumors.

## Supplementary Materials

The following are available online at https://www.mdpi.com/2072-6694/12/4/796/s1: Supplementary Materials; Table S1: List of all cancer sites included to the SMR analysis for patients diagnosed with cancers at 45+ years of age; Table S2: Analysis of risk of death due to Alzheimer’s disease in patients diagnosed with cancers at less than 45 years of age; Table S3: Characteristics of cases of AD deaths in patients of black race diagnosed with cancers at less than 45 years of age; Table S4: Cohort characteristics of breast cancer patients of white race from SEER 13 registry included in the analysis of the influence of chemotherapy on the Alzheimer’s disease death rate; Table S5: Cohort characteristics of breast cancer patients of white race from SEER 13 registry included in the analysis of the influence of radiation therapy on the Alzheimer’s disease death rate.; Table S6: Risk of AD death in breast cancer patients relative to the reference population stratified by chemotherapy status; Table S7: Risk of AD death in breast cancer patients relative to the reference population stratified by radiotherapy status; Table S8: Cohort characteristics of breast cancer patients from SEER 9 registry included in the analysis of the influence of radiotherapy, chemotherapy and demographic/clinical variables on the Alzheimer’s disease death rate; Table S9: Status of strata in Cox model of AD death hazard in women diagnosed with breast cancer at 45+ years of age; Table S10: Status of variable in Cox model of AD death hazard in women diagnosed with breast cancer at 45+ years of age; Table S11: Test for proportionality of hazards violation for the Cox model of AD death hazard in women diagnosed with breast cancer at 45+ years of age; Figure S1: Cumulative hazards ratio of AD death between age groups 5 (85+ years) and 3 (65–74 years); Figure S2: Baseline cumulative hazards of AD death in patients diagnosed with breast cancer at 45+ years versus time since cancer diagnosis, stratified by race; Figures S3–S7: Log(−log survival) curves for AD death in women diagnosed with breast cancer at 45+ years. Cox model with variable race stratified on five 10-year age groups; Figure S8: Baseline cumulative hazard curves for AD death in white women diagnosed with breast cancer at 45+ years. Cox model stratified on chemotherapy status; Figure S9: Log(−log survival) curves for AD death in white women diagnosed with breast cancer at 45+ years. Cox model stratified on chemotherapy status; Figure S10: Baseline cumulative hazard curves for AD death in white women diagnosed with breast cancer at 45+ years. Cox model stratified on radiotherapy status; Figure S11: Log(−log survival) curves for AD death in white women diagnosed with breast cancer at 45+ years. Cox model stratified on radiotherapy status; Figure S12: Cumulative AD mortality ratios (CMRs) for beam radiotherapy versus no/unknown radiation therapy groups.

## Abbreviations

–2LL	–2 log-likelihood statistics
AD	Alzheimer’s disease
AIAN	American Indian/Alaska Native (race)
API	Asian/Pacific Islander (race)
B	Black (race)
BBB	Blood-brain barrier
BC	Breast cancer
CICI	Chemotherapy-induced cognitive impairment
CMR	Cumulative mortality ratio
COD	Cause of death
ICD	International Classification of Diseases
ICD-O-3	International Classification of Diseases for Oncology, 3rd revision
IDC	Invasive ductal carcinoma
IR	Ionizing radiation
LDIR	Low-dose ionizing radiation
LOAD	Late-onset Alzheimer’s Disease
MRRMH	Mortality rate ratio (weighted average by Mantel–Haenszel)
NGS	Next-generation sequencing
NOS	Not otherwise specified
NRF2	Nuclear factor erythroid 2–related factor 2; NFE2L2 gene
O	Number of observed events
PYR	Person-time at risk (years)
SE	Standard error
SEER	Surveillance, Epidemiology and End Results
SMR	Standardized mortality ratio
W	White (race)
WBRT	Whole brain radiotherapy
