# Supplementary material for: Risk of Alzheimer’s Disease in Cancer Patients: Analysis of Mortality Data from the US SEER Population-Based Registries
Source: Cancers (Basel). 2020 Mar 26;12(4):796. doi: 10.3390/cancers12040796 (PMC7226270; doi:10.3390/cancers12040796)
Supplement: Supplementary file 1 [file cancers-12-00796-s001.pdf]

Supplementary Materials: Risk of Alzheimer's Disease in Cancer Patients: Analysis of Mortality Data from the US SEER Population-Based Registries

Roman Mezencev and Yury O. Chernoff

Supplemental Methods and Results

*Section 1. SEER*Stat MP-SIR Session Settings*

Data: Incidence—SEER 9 Regs Custom Data (with additional treatment fields), Research Data, Nov 2018 (1975–2016) for SMR cases (released: April 2019 based on the November 2018 submission) [1].
Rates: U.S. Mortality 1975–2016 (Nov 2018 submission), Race: W/B/O, Event: COD rec (HIV grouped w/oth infections) [2]
Selection: Select Only Malignant Behavior; Exclude Death Certificate Only and Autopsy Only Cases; Multiple Primary Selection: First Primary Only (Sequence Number 0 or 1).
Parameters: Exposure Date: Date of diagnosis recode; Latency exclusion period (months) 0; Cutoff Dates: Start—Jan 1975, End—Dec 2016;
Events: Analysis Type—Single outcome analysis; Exit Point: Exit at Death, Early Exit at Next Malignant Tumor; Event Variable (original)—COD rec (HIV grouped w/oth infections); Events - Alzheimer disease (ICD-9 and ICD-10 only).
Statistic: Confidence Interval—Exact Method

1. Surveillance, Epidemiology, and End Results (SEER) Program (www.seer.cancer.gov) SEER*Stat Database: Incidence—SEER 9 Regs Custom Data (with additional treatment fields), Nov 2018 Sub (1975–2016) for SMRs—Linked To County Attributes—Total U.S., 1969–2017 Counties, National Cancer Institute, DCCPS, Surveillance Research Program, released April 2019, based on the November 2018 submission.
2. U.S. Mortality 1975–2016 (Nov 2018 sub), Race (W/B/O), Event: COD rec (HIV grouped w/oth infectious) Rates are only included for white, black, and other races (not including other unspecified 1978–1991 and unknown). Mortality—All COD, Aggregated Total U.S. (1969–2016) <Katrina/Rita Population Adjustment>

Section 2 Differences Between Black and White Patients Diagnosed with Cervical Cancers at Age ≥ 45 years Recorded in SEER9 Registry Between 01/1975 and 12/2016.

Difference between column means (continuous variables) or proportions (categorical variables) is considered significant for *t*-test or Fisher exact test 2-sided *p*-value < 0.05. For each significant pair, the key of the category with the smaller column proportion appears in the category with the larger column proportion. Significance level for upper case letters (A,B): 0.05 (not adjusted for multiple tests)

Characteristics		Race: Black (A)		Race: White (B)	
		Count	Median	Count	Median
Number of Patients		4096		18443	
Age at diagnosis [years]			60		60
Person Time [years]			3		4
Year of diagnosis			1993		1993
ICD-O-3 Histology/behavior	8000/3: Neoplasm, malignant	29		120	
	8001/3: Tumor cells, malignant	13 B		8	
	8004/3: Malignant tumor, spindle cell type	0 [1]		1	
	8010/3: Carcinoma, NOS	211		838	
	8012/3: Large cell carcinoma, NOS	8		30	
	8013/3: Large cell neuroendocrine carcinoma	2		6	

**Characteristics**	**Race: Black (A)**		**Race: White (B)**	
	**Count**	**Median**	**Count**	**Median**
8015/3: Glassy cell carcinoma	0 [1]		2	
8020/3: Carcinoma, undifferentiated, NOS	13		44	
8021/3: Carcinoma, anaplastic, NOS	9		24	
8022/3: Pleomorphic carcinoma	0 [1]		2	
8032/3: Spindle cell carcinoma, NOS	0 [1]		7	
8041/3: Small cell carcinoma, NOS	24		120	
8042/3: Oat cell carcinoma	0 [1]		2	
8044/3: Small cell carcinoma, intermediate cell	0[1]		1	
8045/3: Combined small cell carcinoma	2		4	
8046/3: Non-small cell carcinoma	1		5	
8050/3: Papillary carcinoma, NOS	1		15	
8051/3: Verrucous carcinoma, NOS	4		22	
8052/3: Papillary squamous cell carcinoma	18		59	
8070/3: Squamous cell carcinoma, NOS	2309 B		9339	
8071/3: Squamous cell carcinoma, keratinizing, NOS	352 B		1308	
8072/3: Squamous cell carcinoma, large cell, nonkeratinizing, NOS	280		1505 A	
8073/3: Squamous cell carcinoma, small cell, nonkeratinizing	18		53	
8074/3: Squamous cell carcinoma, spindle cell	2		6	
8075/3: Squamous cell carcinoma, adenoid	2		6	
8076/3: Squamous cell carcinoma, micro-invasive	148		764	
8078/3: Squamous cell carcinoma with horn formation	1		0[1]	
8082/3: Lymphoepithelial carcinoma	1		2	
8083/3: Basaloid squamous cell carcinoma	5		18	
8084/3: Squamous cell carcinoma, clear cell type	0 [1]		1	
8090/3: Basal cell carcinoma, NOS	1		1	
8092/3: Infiltrating basal cell carcinoma, NOS	1		0[1]	
8098/3: Adenoid basal cell carcinoma	3		8	
8120/3: Transitional cell carcinoma, NOS	0 [1]		4	
8123/3: Basaloid carcinoma	0 [1]		1	
8140/3: Adenocarcinoma, NOS	285		2195 A	
8144/3: Adenocarcinoma, intestinal type	1		6	
8147/3: Basal cell adenocarcinoma	0 [1]		4	
8200/3: Adenoid cystic carcinoma	19 B		23	
8210/3: Adenocarcinoma in adenomatous polyp	1		6	
8240/3: Carcinoid tumor, NOS	0 [1]		1	
8246/3: Neuroendocrine carcinoma, NOS	6		36	
8255/3: Adenocarcinoma with mixed subtypes	1		8	
8260/3: Papillary adenocarcinoma, NOS	22		166 A	
8261/3: Adenocarcinoma in villous adenoma	0 [1]		2	
8262/3: Villous adenocarcinoma	0 [1]		9	
8263/3: Adenocarcinoma in tubulovillous adenoma	0[1]		18	
8310/3: Clear cell adenocarcinoma, NOS	24		149	
8320/3: Granular cell carcinoma	0 [1]		1	
8323/3: Mixed cell adenocarcinoma	13		48	
8380/3: Endometrioid carcinoma	17		190 A	

	Characteristics	Race: Black (A)		Race: White (B)	
		Count	Median	Count	Median
	8382/3: Endometrioid adenocarcinoma, secretory variant	0 [1]		1	
	8384/3: Adenocarcinoma, endocervical type	10		164 A	
	8430/3: Mucoepidermoid carcinoma	1		5	
	8441/3: Serous cystadenocarcinoma, NOS	8		21	
	8460/3: Papillary serous cystadenocarcinoma	9		36	
	8461/3: Serous surface papillary carcinoma	1		4	
	8480/3: Mucinous adenocarcinoma	19		148 A	
	8481/3: Mucin-producing adenocarcinoma	8		65	
	8482/3: Mucinous adenocarcinoma, endocervical type	4		35	
	8490/3: Signet ring cell carcinoma	0 [1]		5	
	8560/3: Adenosquamous carcinoma	127		549	
	8570/3: Adenocarcinoma with squamous metaplasia	1		20	
	8574/3: Adenocarcinoma with neuroendocrine differentiation	0 [1]		3	
	8575/3: Metaplastic carcinoma, NOS	0 [1]		2	
	8720/3: Malignant melanoma, NOS	1		7	
	8770/3: Mixed epithelioid and spindle cell melanoma	0 [1]		1	
	8772/3: Spindle cell melanoma, NOS	0 [1]		1	
	8800/3: Sarcoma, NOS	4		17	
	8801/3: Spindle cell sarcoma	0 [1]		1	
	8802/3: Giant cell sarcoma	0 [1]		1	
	8805/3: Undifferentiated sarcoma	0 [1]		1	
	8810/3: Fibrosarcoma, NOS	1		0[1]	
	8890/3: Leiomyosarcoma, NOS	5		39	
	8891/3: Epithelioid leiomyosarcoma	0 [1]		3	
	8896/3: Myxoid leiomyosarcoma	2		0[1]	
	8900/3: Rhabdomyosarcoma, NOS	2		4	
	8902/3: Mixed type rhabdomyosarcoma	1		0[1]	
	8910/3: Embryonal rhabdomyosarcoma, NOS	0 [1]		2	
	8920/3: Alveolar rhabdomyosarcoma	1		0[1]	
	8933/3: Adenosarcoma	0 [1]		23	
	8935/3: Stromal sarcoma, NOS	1		5	
	8950/3: Mullerian mixed tumor	16 B		32	
	8951/3: Mesodermal mixed tumor	5		8	
	8980/3: Carcinosarcoma, NOS	18 B		32	
	9064/3: Germinoma	0 [1]		1	
	9100/3: Choriocarcinoma, NOS	1		0[1]	
	9110/3: Mesonephroma, malignant	3		17	
	9364/3: Peripheral neuroectodermal tumor	0 [1]		2	
Grade	Moderately differentiated; Grade II	891		4593 A	
	Poorly differentiated; Grade III	1167 B		4814	
	Undifferentiated; anaplastic; Grade IV	131		598	
	Unknown	1722 B		6992	
	Well differentiated; Grade I	185		1446 A	
SEER historic stage A (1973–2015)	Blank(s)	93		366	
	Distant	589 B		2372	
	Localized	1417		7339 A	

	**Characteristics**	**Race: Black (A)**		**Race: White (B)**	
		**Count**	**Median**	**Count**	**Median**
	Regional	1697 B		7175	
	Unstaged	300 B		1191	
Chemotherapy	No	3091		14092	
	Yes	1005		4351	
Radiotherapy	Beam radiation	1123 B		4273	
	Combination of beam with implants or isotopes	1263		6364 A	
	None/Unknown	1314		6503 A	
	Other than beam radiation (1973-1987 cases only)	63		346	
	Radiation, NOS method or source not specified	125 B		302	
	Radioactive implants (includes brachytherapy) (1988+)	121 B		393	
	Radioisotopes (1988+)	2		18	
	Recommended, unknown if administered	63 B		154	
	Refused (1988+)	22		90	

[1] Category not used in comparisons, because its column proportion is equal to 0 or 1.

Section 3 Comparison of Age-Specific Mortality Rates for Alzheimer Disease in Breast Cancer Patients

This analysis included white women diagnosed with breast cancer of IDC histological type at age groups 65–69 years, 70–74 years, 75–79 years, 80–84 years between 2000–2016 (Incidence-SEER 18 Regs excluding AK Research Data, Nov 2018 Sub (2000–2016) for SMR) who attained ages 70–74 years, 75–79 years, 80–85 years, and 85–89 years. Death of Alzheimer disease was considered as an event of interest and cases with any other outcome (diagnosis of second primary cancers, death of other causes, end of study or a loss of follow-up) were censored. Numbers of events and total person-years were accumulated for each age group at diagnosis and group of attained age and the age-specific AD mortality rates were calculated

Results

Age at BC: age at breast cancer diagnosis; O: number of observed AD deaths; SMR: standardized mortality for AD death; PYR: accumulated person-years; Age-specific mortality rates are per 100,000 person-years; CI95: 95%-confidence intervals calculated by the Mid-p-exact method; * $p < 0.05$ (for SMR; not adjusted for multiple tests).

**Attained Age**	**70–74**					**75–79**				
Age at BC	O	SMR	PYR	Age-specific mortality rate	CI95	O	SMR	PYR	Age-specific mortality rate	CI95
65–69	42	0.83	135706.9	30.95	25.59–41.44	81	1.16	57092.65	141.9	113.4–175.4
70–74	19	0.60*	87453.05	21.73	13.47–33.30	103	0.81 *	106995	96.27	78.98–116.3
75–79	N/A	N/A	N/A	N/A	N/A	49	0.59 *	72041.31	68.02	50.87–89.18
80–84	N/A	N/A	N/A	N/A	N/A	N/A	N/A	N/A	N/A	N/A
85–89	N/A	N/A	N/A	N/A	N/A	N/A	N/A	N/A	N/A	N/A
**Attained Age**	**80–84**					**85–89**				
Age at BC	O	SMR	PYR	Age-specific mortality rate	CI95	O	SMR	PYR	Age-specific mortality rate	CI95
65–69	57	1.2	13361.12	426.6	326.1–457.8	2	0.6	237.2	843.2	141.4–278.6

70–74	140	0.96	44074.36	317.6	268.2–373.6	90	0.75 *	9457.58	951.6	769.7–1164
75–79	195	0.71 *	86631.93	225.1	195.1–258.4	285	0.73 *	33044	862.5	766.6–967.1
80–84	113	0.71 *	51503.74	219.4	181.7–262.8	347	0.58 *	53422.58	649.5	583.8–720.6
85–89	N/A	N/A	N/A	N/A	N/A	127	0.43 *	27184.5	467.2	391.1–553.9

Summary of results: Age-specific mortality rates for Alzheimer disease at different ages of breast cancer diagnosis (point estimates). Color coding indicates time between BC diagnosis and attained age (as differences between centers of time intervals: black =0 years; blue = 5 years; green = 10 years; red: 15 years; violet = 20 years).

Attained Age	70–74	75–79	80–84	85 ± 89
Age at BC				
65–69	30.95	141.9	426.6	843.2
70–74	21.73	96.27	317.6	951.6
75–79		68.02	225.1	862.5
80–84			219.4	649.5
85–89				467.2

Section 4 Cox Proportional Hazards Models for AD Death Rate in Women Diagnosed with Breast Cancer at ≥45 Years.

4.1. Analysis for the Effect of Race Stratified on 5 Age Groups: 1 (45–54 years), 2: (55–64 years), 3 (65–74 years), 4 (75–84 years), and 5 (85+ years).

Variable	Estimate	SE	z-value	*p*-value	Hazard Rate (HR)	CI95 (HR)
**Race:AIAN**	–0.358	0.409	–0.877	0.3806	0.699	0.314–1.557
**Race:API**	–0.605	0.090	–6.727	1.73e-11	0.546	0.458–0.651
**Race:B**	–0.292	0.087	–3.372	7.46e-4	0.747	0.631–0.885
**Race:W**	Baseline					
N = 337,267; Number of events: 3,876						
Likelihood ratio test = 65.71 on 3 df, *p* = 4e-14; Wald test = 55.71 on 3 df, *p* = 5e-12						

4.2. Analysis for Effect of Age at BC Diagnosis in White Women Diagnosed at ≥45 Years with Age as Continuous (A) or Ordinal (grouped) Variable (B)

Non proportional hazards detected when age is modeled in five 10-year age groups (B) but not when modelled as a continuous variable

A	Estimate	SE	z-value	*p*-value	Hazard Rate (HR)	CI95 (HR)
**Age at BC diagnosis [yrs.]**	0.1636	2.09e-3	78.17	<2e-16	1.178	1.173–1.183
N = 282,092; Number of events: 3,603						
Likelihood ratio test = 7663 on 1 df, *p* = <2e-16; Wald test = 6111 on 1 df, *p* = <2e-16						
Schoenfeld residuals correlation: rho = –0.0289; Chi-sq = 2.14, *p* = 0.144						
**B**	**Estimate**	**SE**	**z-value**	***p*-value**	**Hazard Rate (HR)**	**CI95 (HR)**
Age at BC diagnosis (45–54 yrs.)	Baseline					
Age at BC diagnosis (55–64 yrs.)	1.738	0.0955	18.2	<2e-16	5.688	4.717–6.858
Age at BC diagnosis (65–74 yrs.)	3.322	0.0936	35.5	<2e-16	27.710	23.066–33.287
Age at BC diagnosis (75–84 yrs.)	4.796	0.0961	49.9	<2e-16	121.024	100.247–146.107
Age at BC diagnosis (85+ yrs.)	5.951	0.1073	55.4	<2e-16	384.220	311.319–474.192
N = 282,092; Number of events: 3,603<br>Likelihood ratio test = 6926 on 4 df, *p* = <2e-16; Wald test= 5198 on 4 df, *p* = <2e-16<br>Schoenfeld residuals correlation:<br>(55–64 yrs.): rho = -0.0829, Chisq = 23.4, *p* = 1.31e-06<br>(65–74 yrs.): rho = -0.1327, Chisq = 56.0, *p* = 7.10e-14<br>(75–84 yrs.): rho =-0.1473, Chisq = 67.3, *p* = 2.28e-16<br>(85+ yrs.): rho =-0.1314, Chisq = 53.9, *p*=2.08e-13<br>GLOBAL: Chisq = 81.9, *p* = 6.79e-17						

4.3. Analysis for Effect of Chemotherapy, Radiation Therapy, Year of Diagnosis and Age at BC Diagnosis in White Women Diagnosed at ≥45 years

Variable	Estimate	SE	z-value	*p*-value	Hazard rate (HR)	CI95 (HR)
Chemotherapy: Yes	−0.1942	0.0657	−2.953	0.00315	0.824	0.724–0.937
Chemotherapy: No/Unknown	Baseline					
Radiation therapy: Beam	−0.1322	0.0382	−3.463	0.00054	0.876	0.813–0.944
Radiation therapy: No/Unknown	Baseline					
Radiation therapy: Other	−0.2963	0.1533	−1.933	0.05323	0.744	0.551–1.004
Age at BC diagnosis	0.1587	0.0022	72.77	<2e−16	1.1720	1.167–1.177
Year at BC diagnosis:1	Baseline					
Year at BC diagnosis:2	0.541	0.0552	9.80	<2e−16	1.717	1.541–1.914
Year at BC diagnosis:3	1.105	0.0611	18.090	<2e−16	3.020	2.679–3.404
Year at BC diagnosis:4	1.369	0.0796	17.199	<2e−16	3.930	3.363–4.594

N = 282,092; Number of events: 3,603 Likelihood ratio test = 8124 on 7 df, *p* = <2e-16; Wald test = 6568 on 7 df, *p* = <2e-16.

Results of a test for the proportional hazards assumption of a Cox regression model using cox.zph function in the R. Rho: correlation coefficient between transformed survival time and the scaled Schoenfeld residuals; *p*-value is two-sided.

Variable	rho	Chisq	*p*-value
Chemotherapy: YES	0.05817	12.60716	3.84e-04
Radiotherapy:Beam	0.047615	8.26627	4.04e-03
Radiotherapy:Other	0.000638	0.001479	9.69e-01
Year at diagnosis 2	−0.094366	30.64294	3.10e-08
Year at diagnosis 3	−0.104237	35.12337	3.09e-09
Year at diagnosis 4	−0.076388	18.98831	1.32e-05
Age at diagnosis (cont)	0.006976	0.12938	7.19e-01
Global	NA	54.66111	1.74e-09

4.4. Analysis for Effect of Chemotherapy, Radiation Therapy and Age at BC Diagnosis (Continuous Variable) in White Women Diagnosed at ≥45 years

Variable	Estimate	SE	z-value	*p*-value	Hazard Rate (HR)	CI95 (HR)
Chemotherapy: Yes	−0.0677	0.0653	−1.04	0.300	0.935	0..822–1.062
Chemotherapy: No/Unknown	Baseline					
Radiation therapy: Beam	0.0932	0.0368	2.53	0.011	1.098	1.021–1.180
Radiation therapy: No/Unknown	Baseline					
Radiation therapy: Other	−0.0353	0.1524	−0.23	0.817	0.965	0.716–1.301
Age at BC diagnosis	0.1638	0.00217	75.38	<2e-16	1.180	1.173–1.183

N = 282,092; Number of events: 3,603 Likelihood ratio test= 7671 on 4 df, *p* = <2e-16; Wald test = 6089 on 4 df, *p* = <2e-16.

Results of a test for the proportional hazards assumption of a Cox regression model using cox.zph function in the R. Rho: correlation coefficient between transformed survival time and the scaled Schoenfeld residuals; *p*-value is two-sided.

Variable	rho	Chisq	*p*-value
Chemotherapy: YES	0.03769	5.173	0.0229
Radiotherapy:Beam	0.00974	0.338	0.5609
Radiotherapy:Other	−0.01249	0.563	0.4531
Age at diagnosis (cont)	−0.01796	0.859	0.3540
Global	NA	8.707	0.0688

Removal of the variable "Year at diagnosis" produced a model predicting significantly increased risk of the AD death in breast cancer patients treated with beam radiation relative to breast cancer patients with no beam radiation therapy, adjusted for age at diagnosis (continuous) and the use of chemotherapy (HR = 1.10; CI95: 1.02–1.18; *p* = 0.011). Similarly, administration of

chemotherapy was associated with decreased risk of AD death relative to the group with no or unknown chemotherapy administration, but the risk reduction did not reach statistical significance (HR = 0.94; CI95: 0.82–1.06; $p = 0.300$). Risk of AD death was significantly increasing with increasing age at breast cancer (estimated 18% increase in the expected hazard per one-year increase in the age at diagnosis). Violation of proportionality assumption was found (Global and Chemotherapy: Yes).

Section 5 Analysis of the Role of Estrogen Receptor (ER)-Status in Younger Breast Cancer (BC) Patients (age at Diagnosis < 54 years) on the Risk of Alzheimer Disease Death

This analysis was performed using cases recorded in SEER 13 registry for SMR (1992–2016) for all races combined.

Time Since BC Diagnosis (months)	**ER-positive BC**			**ER-negative BC**		
	O	SMR (CI95)	N	O	SMR (CI95)	N
2–11	0	0.00 (0.00–19.12)	130,855	0	0.00 (0.00–67.18)	45,918
12–59	0	0.00 (0.00–2.51)	121,899	0	0.00 (0.00–9.33)	42,639
60–119	4	1.12 (0.31–2.87)	86,182	2	1.99 (0.24–7.19)	27,912
120+	20	1.02 (0.62–1.58)	50,934	5	0.73 (0.24–1.71)	18,328
Total	24	0.97 (0.62–1.44)	130,855	7	0.85 (0.34–1.75)	45,918

O: number of observed AD deaths; SMR: standardized mortality ratio for AD death; CI95: 95%-confidence intervals calculated by the Mid-p-exact method; * $p < 0.05$ (for SMR, *not adjusted for multiple tests)*, *N*: number of BC cases.

Table S1. List of all cancer sites included to the SMR analysis for patients diagnosed with cancers at ≥ 45 years of age. The table conveys aggregation of cancer sites used in the SMR analysis. The rows highlighted by grey color correspond to cancer sites for which the total number of person-years at risk did not reach 100,000 person-years for any specific race. O: number of deaths due to Alzheimer disease in a group of patients with specific race who were diagnosed with cancers at specific sites. SMR: standardized mortality ratios for AD deaths in patients diagnosed with cancer relative to the general populations; PYR: accumulated person-years at risk. Races: AIAN: American Indian/Alaska Native; API: Asian/Pacific Islander. * $p < 0.05$ for SMR values *(not adjusted for multiple tests).*

Cancer Site	White			Black			AIAN			API		
	O	SMR	PYR	O	SMR	PYR	O	SMR	PYR	O	SMR	PYR
All Sites	21,098	0.97 *	17,226,235.60	1,086	0.98	1,547,446.29	40	1.38	81,733.97	855	1.23 *	1,166,766.56
All Solid Tumors	19,868	0.99 *	15,943,508.31	1,037	0.99	1,442,908.75	40	1.46 *	75,722.22	810	1.23 *	1,095,727.10
All Lymphatic and Hematopoietic Diseases	960	0.75 *	1,125,432.10	36	0.68 *	91,785.13	0	0	5,121.82	35	1.05	62,056.49
Oral cavity	319	1.02	375,369.60	11	1.2	24,700.24	2	3.94	1,524.91	9	1	18,163.39
Pharynx	12	0.73	37,676.06	2	1.69	5,252.56	0	0	252.28	5	2.1	13,331.29
Esophagus	39	0.91	52,094.20	3	1.37	6,912.72	0	0	266.6	0	0	3,096.08
Stomach	133	0.93	115,903.55	16	1.02	19,615.24	0	0	1,214.15	29	1.35	31,539.76
Small Intestine	39	0.74	48,794.97	4	0.92	7,264.77	0	0	246.56	4	3.56	2,961.59
Colon excluding Rectum	2,435	0.99	1,397,529.18	141	1	138,443.43	5	1.69	6,040.03	129	1.33 *	120,446.85
Rectum and Rectosigmoid Junction	781	1.01	620,514.43	42	1.32	47,469.32	1	1	3,285.69	47	1.35	64,251.65
Anus, Anal Canal and Anorectum	54	0.95	52,094.25	2	0.61	5,549.41	0	0	240.09	3	2.46	1,881.47
Liver	5	0.26 *	34,398.00	2	1.19	6,415.47	0	0	833.87	7	1.6	13,770.56
Gallbladder	31	1.41	14,141.80	2	2.34	1,341.05	0	0	470.25	2	1.58	1,941.92
Pancreas	46	0.77	65,098.37	6	1.23	8,820.35	0	0	442.46	5	1.53	6,312.29
Nose, Nasal Cavity and Middle Ear	29	1.31	22,864.98	1	1.2	1,752.72	0	0	201.58	2	2.84	2,299.17
Larynx	105	0.80 *	188,869.85	11	1.27	22,761.68	0	0	574.6	3	0.88	7,879.55
Pleura	0	0	395.78	0	0	45.05	0	0	7.75	0	0	44.01
Lung and Bronchus	553	0.84 *	743,630.00	42	1.13	83,617.89	3	3.44	2,896.29	25	0.94	55,754.13
Bones and Joints	12	0.89	18,686.80	1	1.87	1,331.15	0	0	130.8	0	0	980.71
Soft Tissue including Heart	85	0.92	81,528.15	2	0.43	8,283.67	2	8.39 *	469.32	5	1.33	6,472.18
Melanoma of the Skin	814	0.92 *	853,069.15	5	1.67	3,441.66	0	0	1,582.13	3	1.01	5,322.96
Female Breast	5,375	1.01	3,847,928.29	229	0.98	300,647.12	9	1.45	18,394.56	179	1.12	285,072.71
Cervix Uteri	102	0.93	147,863.25	25	1.56*	27,857.60	0	0	2,079.22	12	1.31	22,348.09
Corpus Uteri	1,577	1.02	1,057,883.80	35	0.94	46,473.50	2	1.34	4,299.48	32	1.05	64,136.37
Ovary	185	0.82 *	228,702.81	9	1.06	13,223.97	1	2.55	1,788.23	5	0.75	17,139.28
Prostate	4,914	1.01	3,851,028.54	372	0.92	536,227.59	10	1.38	16,580.77	207	1.22 *	222,993.15
Testis	4	0.36 *	46,310.76	0	0	1,039.40	0	0	208.07	0	0	1,693.35
Urinary Bladder	1,307	0.95 *	991,275.67	30	0.89	35,724.41	3	4	2,208.48	46	1.47 *	38,391.77
Kidney	343	0.95	372,034.87	19	0.85	39,159.80	1	0.68	4,812.42	15	1.42	22,663.97
Renal Pelvis	41	1.18	26,209.00	2	2.53	1,105.84	0	0	69.88	0	0	1,752.16
Ureter	17	1.01	13,449.13	0	0	327.92	0	0	16.42	1	0.98	1,257.19
Brain	23	1.47	52,077.42	2	2.49	2,785.12	0	0	229.27	4	8.03 *	2,712.95
Cranial Nerves Other Nervous System	9	1.25	10,459.26	0	0	1,063.93	1	26.95	132.4	1	3.88	823.37

Cancer Site	White			Black			AIAN			API		
	**O**	**SMR**	**PYR**	**O**	**SMR**	**PYR**	**O**	**SMR**	**PYR**	**O**	**SMR**	**PYR**
Thyroid	170	0.78 *	309,954.66	5	0.53	21,156.47	0	0	2,490.74	10	0.77	40,503.79
Thymus	3	0.62	7,283.73	0	0	1,259.73	0	0	52.35	0	0	1,919.17
Adrenal Gland	1	0.47	3,520.86	1	12.77	400.41	0	0	34.85	0	0	321.16
Hodgkin Lymphoma	16	0.50 *	54,416.88	2	1.61	4,999.99	0	0	211.02	0	0	1,903.80
Non-Hodgkin Lymphoma	559	0.77 *	616,708.96	17	0.76	38,698.18	0	0	2,647.04	29	1.23	39,848.34
Myeloma	76	0.69 *	121,198.45	10	0.64	28,121.92	0	0	1,085.24	4	1.03	8,877.77
Acute Lymphocytic Leukemia	5	2.31	5,045.63	1	6.47	372.09	0	0	75.82	0	0	612.39
Chronic Lymphocytic Leukemia	245	0.77 *	230,998.71	4	0.39 *	12,363.99	0	0	562.53	2	0.64	4,372.66
Acute Non-Lymphocytic Leukemia (ANLL)	7	0.48 *	29,746.48	0	0	2,185.63	0	0	218.36	0	0	2,955.75
Chronic Myeloid Leukemia	27	0.87	35,167.76	1	0.71	3,427.15	0	0	209.19	0	0	2,334.80
Mesothelioma	3	0.38	8,937.60	0	0	448.93	0	0	70.81	0	0	359.32
Kaposi Sarcoma	25	1.34	16,845.87	2	2.4	2,192.57	0	0	149.13	0	0	573.29

Table S2. Analysis of risk of death due to Alzheimer disease in patients diagnosed with cancers at the age < 45 years.Results are shown for cancer sites with at least 100,000 person-years for at least one race. O: number of observed AD deaths; SMR: standardized mortality ratio; *N*: number of people in the cohort. Color coding: orange—SMR is statistically significant ($p < 0.05$; not adjusted for multiple tests) and higher than 1.

Cancer Site	Time	White			Black			American Indian /Alaska Native			Asian /Pacific Islander		
		O	SMR	N	O	SMR	N	O	SMR	N	O	SMR	Persons
All Sites	0–11	0	0	347,583	1	242.41	47,745	0	0	3,970	0	0	33,787
	12–59	1	3.37	300,524	1	50.42	37,494	0	0	3,255	0	0	27,994
	60–119	2	2.49	220,198	0	0	23,602	0	0	2,184	1	49.98	18,492
	120+	73	0.97	165,831	8	1.85	16,051	0	0	1,454	0	0	12,537
	Total	76	0.99	347,583	10	2.28	47,745	0	0	3,970	1	0.52	33,787
All Solid Tumors	0–11	0	0	285,780	1	284.18	37,730	0	0	3,280	0	0	28,243
	12–59	0	0	249,476	1	58.83	29,985	0	0	2,723	0	0	23,626
	60–119	2	2.77	182,911	0	0	18,691	0	0	1,844	1	53.63	15,661
	120+	68	0.96	137,735	7	1.75	12,738	0	0	1,245	0	0	10,630
	Total	70	0.97	285,780	9	2.21	37,730	0	0	3,280	1	0.55	28,243
All Lymphatic and Hematopoietic Diseases	0–11	0	0	56,254	0	0	8,699	0	0	615	0	0	4,947
	12–59	1	36.29	47,166	0	0	6,673	0	0	481	0	0	3,999
	60–119	0	0	34,426	0	0	4,299	0	0	304	0	0	2,561
	120+	5	1.34	25,749	1	5.09	2,827	0	0	179	0	0	1,714
	Total	6	1.57	56,254	1	4.89	8,699	0	0	615	0	0	4,947
Melanoma of the Skin	0–11	0	0	39,871	0	0	168	0	0	105	0	0	332
	12–59	0	0	36,819	0	0	144	0	0	92	0	0	297
	60–119	0	0	29,888	0	0	108	0	0	69	0	0	211
	120+	9	0.9	23,333	0	0	88	0	0	51	0	0	160

**Cancer Site**	Time	**White**			**Black**			**American Indian /Alaska Native**			**Asian /Pacific Islander**		
		O	SMR	N	O	SMR	N	O	SMR	N	O	SMR	Persons
	Total	9	0.89	39,871	0	0	168	0	0	105	0	0	332
Female Breast	0–11	0	0	60,747	0	0	10,621	0	0	602	0	0	7,527
	12–59	0	0	56,968	0	0	9,649	0	0	553	0	0	6,919
	60–119	0	0	42,375	0	0	6,054	0	0	380	0	0	4,801
	120+	23	0.95	30,482	4	2.84	3,888	0	0	240	0	0	3,093
	Total	23	0.93	60,747	4	2.79	10,621	0	0	602	0	0	7,527
Cervix Uteri	0–11	0	0	14,463	0	0	2,530	0	0	265	0	0	1,379
	12–59	0	0	13,203	0	0	2,230	0	0	234	0	0	1,218
	60–119	0	0	10,401	0	0	1,595	0	0	170	0	0	872
	120+	6	1.11	8,614	1	1.38	1,313	0	0	135	0	0	671
	Total	6	1.1	14,463	1	1.37	2,530	0	0	265	0	0	1,379
Testis	0–11	0	0	20,980	0	0	574	0	0	256	0	0	962
	12–59	0	0	19,542	1	5,075.03	510	0	0	230	0	0	870
	60–119	1	33.25	16,384	0	0	401	0	0	169	0	0	655
	120+	2	0.78	13,297	0	0	301	0	0	122	0	0	494
	Total	3	1.15	20,980	1	26.17	574	0	0	256	0	0	962
Brain	0–11	0	0	16,745	0	0	1,746	0	0	174	0	0	1,230
	12–59	0	0	13,777	0	0	1,366	0	0	135	0	0	982
	60–119	1	92.82	8,586	0	0	806	0	0	78	0	0	577
	120+	2	7.06	5,863	0	0	551	0	0	53	0	0	374
	Total	3	10	16,745	0	0	1,746	0	0	174	0	0	1,230
Thyroid	0–11	0	0	30,643	0	0	2,157	0	0	367	0	0	4,098
	12–59	0	0	28,680	0	0	1,984	0	0	335	0	0	3,750
	60–119	0	0	22,984	0	0	1,520	0	0	257	0	0	2,832
	120+	7	1.05	16,989	0	0	1,030	0	0	171	0	0	1,999
	Total	7	1.04	30,643	0	0	2,157	0	0	367	0	0	4,098
Hodgkin Lymphoma	0–11	0	0	16,455	0	0	2,138	0	0	74	0	0	784
	12–59	0	0	15,583	0	0	1,931	0	0	62	0	0	724
	60–119	0	0	12,927	0	0	1,433	0	0	49	0	0	529
	120+	1	0.96	10,376	0	0	1,031	0	0	30	0	0	351
	Total	1	0.94	16,455	0	0	2,138	0	0	74	0	0	784
Non-Hodgkin Lymphoma	0–11	0	0	19,467	0	0	3,475	0	0	174	0	0	1,782
	12–59	1	70.57	15,135	0	0	2,372	0	0	125	0	0	1,411
	60–119	0	0	11,090	0	0	1,567	0	0	86	0	0	943
	120+	4	1.95	8,080	1	9.53	1,007	0	0	46	0	0	642
	Total	5	2.38	19,467	1	9.19	3,475	0	0	174	0	0	1,782
Acute Lymphocytic Leukemia	0–11	0	0	8,941	0	0	851	0	0	188	0	0	1,023
	12–59	0	0	7,795	0	0	715	0	0	154	0	0	872

Cancer Site	Time	White			Black			American Indian /Alaska Native			Asian /Pacific Islander		
		O	SMR	N	O	SMR	N	O	SMR	N	O	SMR	Persons
	60–119	0	0	5,423	0	0	440	0	0	92	0	0	578
	120+	0	0	4,056	0	0	305	0	0	67	0	0	415
	Total	0	0	8,941	0	0	851	0	0	188	0	0	1,023

Table S3. Characteristics of cases of AD deaths in patients of black race diagnosed with cancers at < 45 years of age.

Number of Cases		Female	Male
		7	3
Primary cancer site	Stomach	0	1
	Colon	1	0
	Breast	4	0
	Cervix uteri	1	0
	Testis	0	1
	Spinal cord	1	
	Lymphoma		1
Radiation therapy	Beam radiation	1	2
	None/Unknown	6	1
Chemotherapy	No/Unknown	7	1
	Yes	0	2
Survival [months]: Median (range)		363 (242–439)	12 (0–248)
Age at AD death [months] Median (range)		839 (646–907)	443 (344–739)
Age at cancer diagnosis Median (range)		37 (28–44)	36 (27–40)
Year of cancer diagnosis Median (range)		1981 (1976–1993)	2007 (1982–2008)
Time between cancer diagnosis and AD death [months] Median (range)		364 (243–440)	12 (1–249)

Table S4. Cohort characteristics of breast cancer patients of white race from SEER 13 registry included in the analysis of the influence of chemotherapy on the Alzheimer disease death rate.

Age at Cancer Diagnosis [years]	Chemotherapy Status	O	Persons	PYR [years]	Mean PYR [years]	Mean Age at BC Diagnosis [years]	Mean Year of BC Diagnosis	Mean Age at AD Death [years]	Mean Year of AD Death
65–69	No/Unknown	262	24,703	204,284.32	8.27	67.46	2004.74	82.62	2011.95
	Yes	47	10,286	67,058.92	6.52	67.28	2006.84	82.1	2012.55

70–74	No/Unknown	514	23,885	188,234.58	7.88	72.47	2003.78	86.25	2011.27
	Yes	57	6,182	36,944.21	5.98	72.28	2006.56	85.69	2012.33
75–79	No/Unknown	635	21,724	151,796.92	6.99	77.44	2003.55	88.09	2010.16
	Yes	42	3,191	16,912.76	5.3	77.16	2006.23	87.56	2011.95
80–84	No/Unknown	633	16,205	91,108.44	5.62	82.33	2003.83	90.59	2009.38
	Yes	21	1,179	5,000.25	4.24	82.05	2006.41	90.86	2009.2
85–89	No/Unknown	362	9,267	39,982.07	4.31	87.16	2004.38	93.14	2008.77
	Yes	5	293	855.45	2.92	86.96	2007.29	93.07	2008.79
90–94	No/Unknown	110	3,261	9,906.86	3.04	92.02	2004.88	96.47	2008.66
	Yes	0	57	124.03	2.18	91.72	2006.6	-	-
95–99	No/Unknown	25	640	1,515.16	2.37	96.72	2005.3	99.2	2008.23
	Yes	0	8	25.25	3.16	96.42	2004.23	-	-

Table S5. Cohort characteristics of breast cancer patients of white race from SEER 13 registry included in the analysis of the influence of radiation therapy on the Alzheimer disease death rate. O: number of AD deaths in the cohort, *N*: number of BC patients in the cohort; PYR: accumulated person-years at risk, BC: breast cancer, AD: Alzheimer disease.

Age at Cancer Diagnosis [years]	Radiation Therapy Status	O	N	PYR [years]	Mean PYR [years]	Mean Age at BC Diagnosis [years]	Mean Year of BC Diagnosis	Mean Age at AD Death [years]	Mean Year of AD Death
65–69	Beam	143	18,787	147,905.62	7.87	67.4	2006.1	82.5	2012.4
	No	160	14,420	113,268.77	7.85	67.43	2004.0	82.69	2011.7
	Other	6	1,782	10,168.85	5.71	67.38	2009.2	79.68	2011.3
70–74	Beam	259	14,888	115,162.63	7.74	72.4	2004.9	86.63	2012.1
	No	300	13,900	102,957.78	7.41	72.47	2003.3	85.91	2010.7
	Other	12	1,279	7,058.37	5.52	72.42	2009.0	83.91	2013.0
75–79	Beam	294	10,769	78,843.06	7.32	77.35	2004.5	88.45	2010.9
	No	370	13,140	84,507.88	6.43	77.45	2003.1	87.79	2009.7
	Other	13	1,006	5,358.74	5.33	77.3	2008.3	86.88	2013.1
80–84	Beam	217	5,897	36,808.80	6.24	82.17	2004.7	91.69	2010.7
	No	421	10,963	56,738.00	5.18	82.39	2003.4	90.05	2008.6
	Other	16	524	2,561.89	4.89	82.22	2007.8	90.45	2012.4
85–89	Beam	58	1,948	10,109.72	5.19	86.89	2005.2	94.79	2010.4
	No	304	7,358	29,711.60	4.04	87.23	2004.2	92.81	2008.4
	Other	5	254	1,016.19	4	86.84	2007.3	93.85	2012.6
90–94	Beam	10	329	1,193.49	3.63	91.72	2005.6	97.23	2009.2
	No	99	2,943	8,678.73	2.95	92.06	2004.8	96.37	2008.5
	Other	1	46	158.68	3.45	91.44	2006.9	98.9	2016.5
95–99	Beam	1	26	52.9	2.03	96.39	2008.6	98.42	2012.6
	No	24	619	1,482.85	2.4	96.72	2005.1	99.23	2008.0
	Other	0	3	4.66	1.55	97.67	2007.2		

Table S6. Risk of AD death in breast cancer patients relative to the reference population stratified by chemotherapy status. Cohort includes white female breast cancer patients from SEER 13 registry diagnosed at 65+ years (5-year age groups). Data shown for age groups that accumulated at least 100,000 person-years in at least one stratum. O: number of AD deaths in the cohort, SMR: standardized mortality rate (CJ Lower and CI upper CI95 lower and upper bound CI95 values, respectively); N: number of BC patients in the cohort; PYR: accumulated person-years at risk; BC: breast cancer. Color coding: Significantly decreased (green) or increased (orange) risk at $p < 0.05$ (not adjusted for multiple tests).

Age Group [Years]	Time since BC Diagnosis [Months]	Chemotherapy status: No/Unknown						Chemotherapy Status: Yes					
		O	SMR	CI Lower	CI Upper	N	PYR	O	SMR	CI Lower	CI Upper	N	PYR
65–69	0–5	1	0.83	0.02	4.64	24,703	11,750.58	0	0	0	7.05	10,286	4,866.55
	6–11	1	0.73	0.02	4.04	22,937	11,189.02	0	0	0	6.45	9,435	4,555.06
	12–59	10	0.62	0.3	1.15	21,925	73,196.83	3	0.48	0.1	1.41	8,870	27,540.52
	60–119	45	1.13	0.82	1.51	15,001	58,042.36	7	0.55	0.22	1.13	5,216	18,692.75
	120–179	58	0.87	0.66	1.13	8,748	32,535.49	14	0.83	0.45	1.39	2,541	8,484.71
	180–239	95	0.98	0.8	1.2	4,550	14,550.83	14	0.88	0.48	1.47	1,000	2,547.25
	240+	52	1.35 *	1.01	1.77	1,511	3,019.21	9	1.89	0.87	3.59	184	372.09
	Total	262	1.01	0.89	1.14	24,703	204,284.32	47	0.82	0.6	1.08	10,286	67,058.92
70–74	0–5	0	0	0	1.03	23,885	11,349.30	0	0	0	3.65	6,182	2,899.46
	6–11	1	0.25	0.01	1.37	22,118	10,806.14	0	0	0	3.38	5,593	2,692.41
	12–59	27	0.57 *	0.37	0.82	21,204	70,212.48	1	0.09 *	0	0.5	5,224	15,751.00
	60–119	99	0.94	0.77	1.15	14,379	55,013.48	13	0.68	0.36	1.16	2,912	10,126.74
	120–179	172	0.93	0.79	1.07	8,111	28,738.00	26	1.01	0.66	1.48	1,310	4,170.10
	180–239	173	1.40 *	1.2	1.62	3,696	10,546.58	10	0.7	0.33	1.28	463	1,187.94
	240+	42	2.12*	1.53	2.86	853	1,568.60	7	4.65 *	1.87	9.59	75	116.57
	Total	514	1.05	0.96	1.14	23,885	188,234.58	57	0.77 *	0.58	1	6,182	36,944.21
75–79	0–5	2	0.20 *	0.02	0.74	21,724	10,251.39	0	0	0	2.32	3,191	1,494.92
	6–11	7	0.65	0.26	1.34	19,947	9,703.96	0	0	0	2.24	2,878	1,373.10
	12–59	76	0.65 *	0.51	0.82	19,031	62,424.94	7	0.48 *	0.19	0.98	2,648	7,629.28
	60–119	211	0.78 *	0.68	0.89	12,437	45,121.36	12	0.46 *	0.24	0.81	1,339	4,517.46
	120–179	225	1.06	0.92	1.2	6,007	19,036.49	15	0.81	0.45	1.34	549	1,591.39
	180–239	102	1.78 *	1.45	2.16	2,063	4,869.26	7	2	0.8	4.11	144	288.12
	240+	12	2.43 *	1.25	4.24	266	389.51	1	4.36	0.11	24.31	15	18.49
	Total	635	0.93	0.86	1	21,724	151,796.92	42	0.64 *	0.46	0.86	3,191	16,912.76

Table S7. Risk of AD death in breast cancer patients relative to the reference population stratified by radiotherapy status (group "other" was not included). Cohort includes white female breast cancer patients from SEER 13 registry diagnosed at 65+ years (5-year age groups). Data shown for age groups that accumulated at least 100,000 person-years in at least one stratum. O: number of AD deaths in the cohort, SMR: standardized mortality rate (CJ Lower and CI upper CI95 lower and upper bound CI95 values, respectively); N: number of BC patients in the cohort; PYR: accumulated person-years at risk; BC: breast cancer. Color coding: Significantly decreased (green) or increased (orange) risk at $p < 0.05$ (not adjusted for multiple tests).

Age Group [Years]	Time since BC Diagnosis [months]	Radiotherapy: Beam						Radiotherapy: No/Unknown					
		O	SMR	CI Lower	CI Upper	N	PYR	O	SMR	CI Lower	CI Upper	N	PYR
65–69	0–5	0	0	0	3.84	18,787	9,050.61	1	1.5	0.04	8.36	14,420	6,718.96
	6–11	0	0	0	3.38	17,661	8,608.29	1	1.33	0.03	7.41	13,065	6,342.48
	12–59	5	0.40 *	0.13	0.94	16,829	55,232.62	8	0.92	0.4	1.82	12,424	40,822.51
	60–119	27	0.92	0.61	1.34	11,073	42,255.52	24	1.12	0.72	1.67	8,287	31,753.75
	120–179	33	0.72	0.5	1.01	6,244	22,442.74	35	0.97	0.67	1.35	4,742	17,745.14
	180–239	54	0.94	0.7	1.22	2,979	8,838.28	54	1.01	0.76	1.32	2,483	8,011.01
	240+	24	1.26	0.81	1.88	808	1,477.56	37	1.56 *	1.1	2.14	869	1,874.91
	Total	143	0.86	0.73	1.01	18,787	147,905.62	160	1.1	0.94	1.29	14,420	113,268.77
70–74	0–5	0	0	0	1.56	14,888	7,172.66	0	0	0	1.85	13,900	6,472.39
	6–11	0	0	0	1.38	14,003	6,837.75	1	0.45	0.01	2.49	12,548	6,099.83
	12–59	6	0.19 *	0.07	0.42	13,377	44,130.31	22	0.87	0.55	1.32	11,956	38,534.77
	60–119	46	0.71 *	0.52	0.94	8,945	33,670.93	59	1.06	0.81	1.37	7,744	29,617.35
	120–179	95	0.87	0.7	1.06	4,866	16,879.83	101	1.02	0.83	1.24	4,363	15,445.19
	180–239	95	1.39 *	1.12	1.7	2,101	5,761.97	85	1.25 *	1	1.55	1,990	5,825.90
	240+	17	1.88 *	1.1	3.01	418	709.18	32	2.63 *	1.8	3.72	503	962.35
	Total	259	0.9	0.79	1.02	14,888	115,162.63	300	1.14 *	1.01	1.27	13,900	102,957.78

Table S8. Cohort characteristics of breast cancer patients from SEER 9 registry included in the analysis of the influence of radiotherapy, chemotherapy and demographic/clinical variables on the Alzheimer disease death rate. O: number of AD deaths in the cohort, N: number of BC patients in the cohort; PYR: accumulated person-years at risk, BC: breast cancer, AD: Alzheimer disease.

Characteristics		AIAN	API	B	W
Total number of tumors for patient	1	1930	26924	31423	275823
	2–14	237	3992	4765	50897
Chemotherapy	None/Unknown	1138	18034	19364	220038
	Yes	1029	12882	16824	106682
Radiation therapy	Beam	1012	15902	16421	142472
	None/Unknown	1066	14395	18121	173972
	Other	89	619	1646	10276
Age at diagnosis [years]		55 (24–103)	56 (18–104)	56 (18–107)	61 (15–107)

Year of diagnosis: median (range)	2005 (1975–2016)	2005 (1975–2016)	2002 (1975–2016)	1998 (1975–2016)
Year of last follow-up: median (range)	2016 (1979–2016)	2016 (1975–2016)	2014 (1975–2016)	2013 (1975–2016)
PTY [years]	5.88 (0.08–40.33)	6.80 (0.08–41.80)	5.30 (0.08–41.88)	7.55 (0.08–41.88)
Number of AD deaths	6	128	141	3619
Age at AD death [months] Median (range)	1008 (846–1113)	1080 (786–1245)	1054 (724–1264)	1064 (679–1274)
Time between BC and AD death [months]	199 (23–318)		176 (3–459)	160 (2–497)

Table S9. Status of strata in Cox model of AD death hazard in women diagnosed with breast cancer at ≥ 45 years of age. The model was stratified on age at cancer diagnosis and used race as a single variable.

Stratum (Age at diagnosis)	Event	Censored	Censored [%]
1 (45–54 yrs.)	158	55868	99.7%
2 (55–64 yrs.)	594	95002	99.4%
3 (65–74 yrs.)	1277	80557	98.4%
4 (75–84 yrs.)	1406	50520	97.3%
5 (85+ yrs.)	441	15496	97.2%
Total	3876	297443	98.7%

Table S10. Status of variable in Cox model of AD death hazard in women diagnosed with breast cancer at ≥ 45 years of age. The model was stratified on age at cancer diagnosis and used race as a single variable.

Race Variable (levels)	Event	Censored	Censored [%]
AIAN	6	1388	99.6%
API	128	21989	99.4%
B	139	28036	99.5%
W	3603	278489	98.7%
Total	3876	329902	98.8%

Table S11. Test for proportionality of hazards violation for Cox model of AD death hazard in women diagnosed with breast cancer at ≥ 45 years of age. The model was stratified on age at cancer diagnosis and used race as a single variable. Rho: correlation coefficient for scaled Schoenfeld vs log(time).

Variable	Rho	Chisq	*p*-value
Race:AIAN	0.01390	0.749	0.3869
Race:API	–0.00514	0.103	0.7487
Race:B	0.03252	4.099	0.0429
Global	N/A	4.979	0.1733

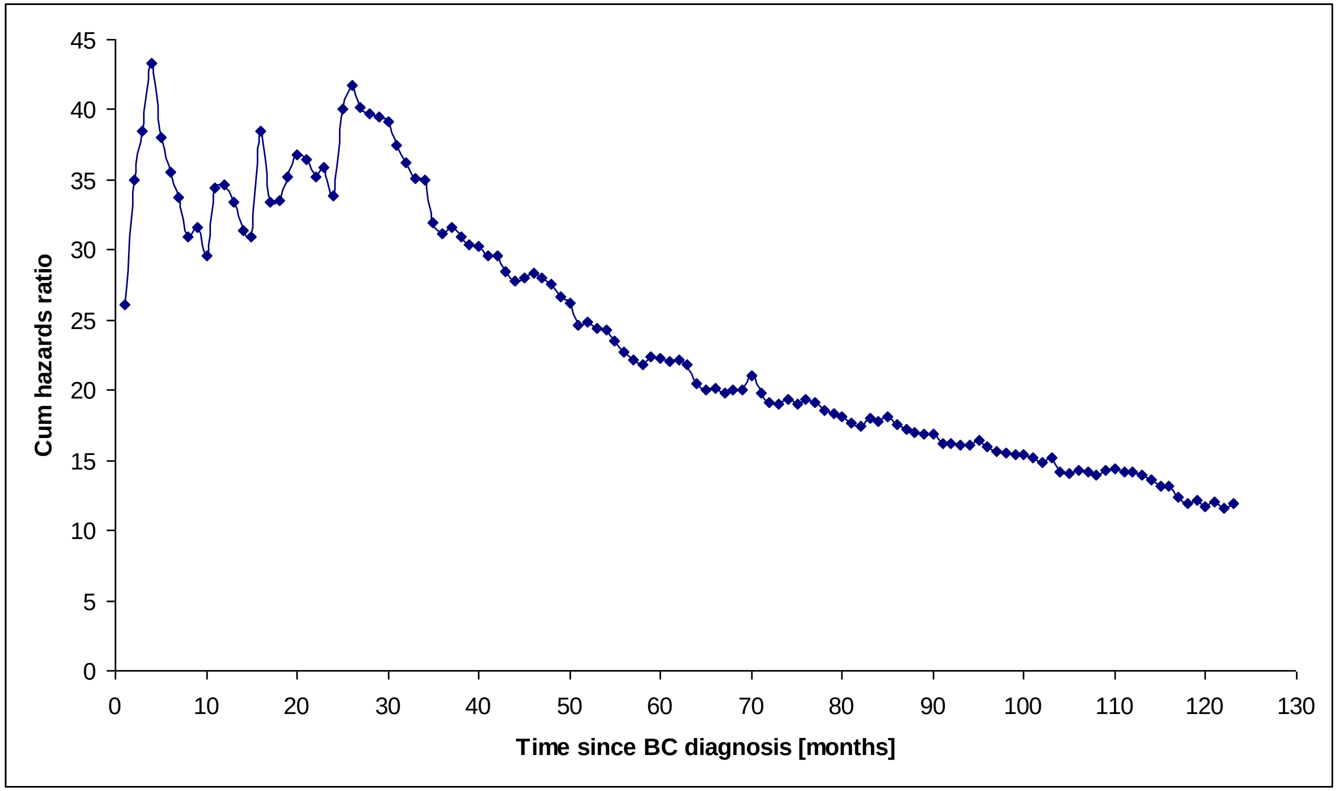

Figure S1. Cumulative hazards ratio of AD death between age groups 5 (85+ years) and 3 (65–74 years). Determined for women of all races diagnosed with breast cancers at ≥45 years of age.

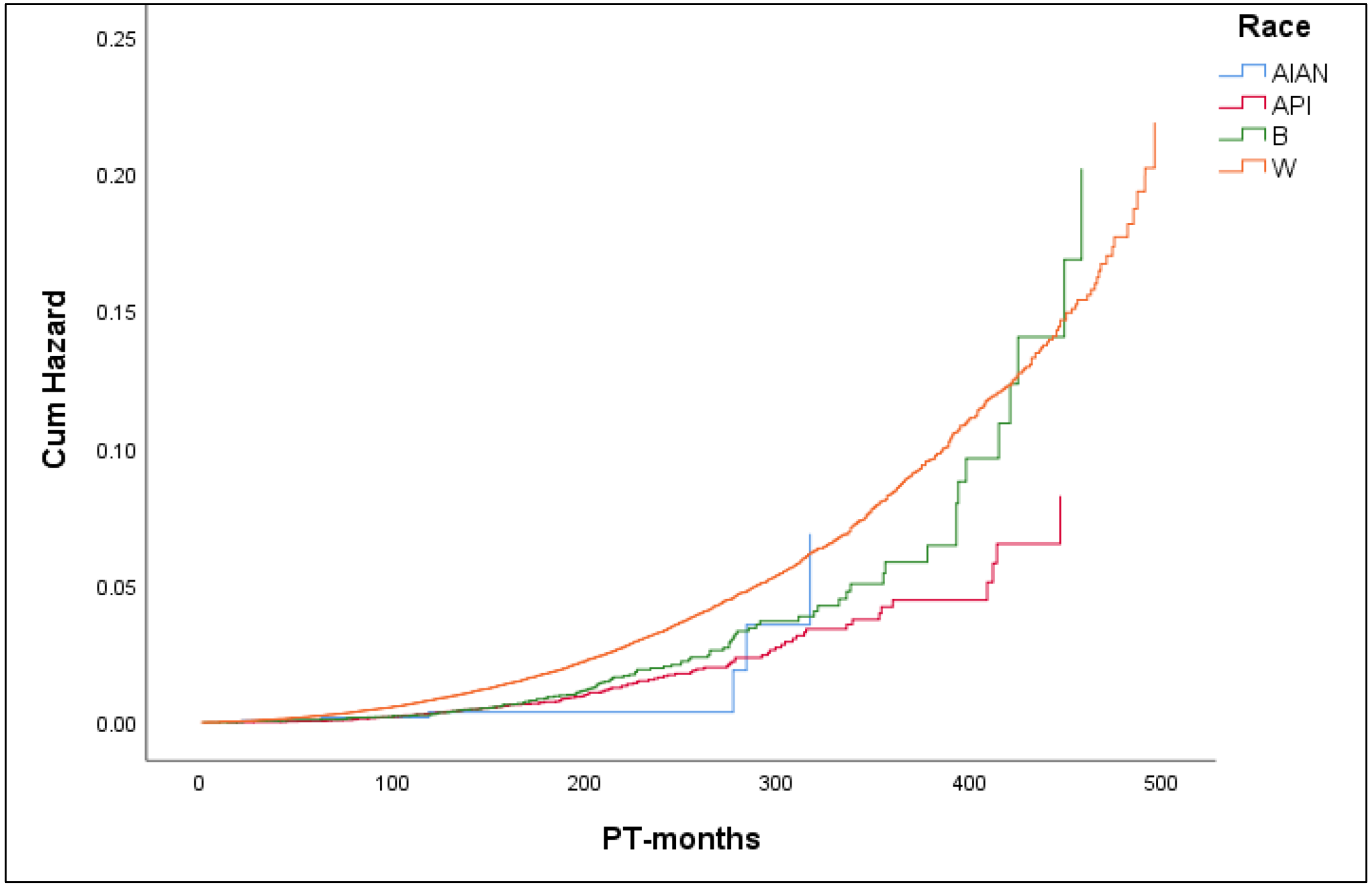

Figure S2. Baseline cumulative hazards of AD death in patients diagnosed with breast cancer at ≥ 45 years vs. time since cancer diagnosis (PT-months), stratified by race: AIAN (American Indian/Alaska Native), API (Asian/Pacific Islander), B (black), W (white).

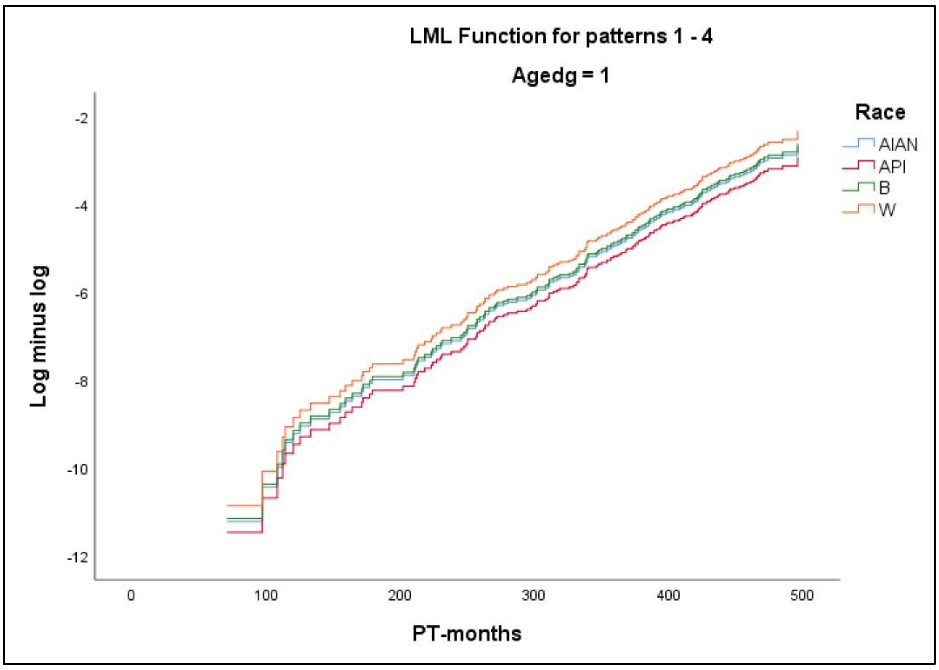

Figure S3. Log(–log survival) curves for AD death in women diagnosed with breast cancer at 45+ years. Cox model with variable race stratified on five 10-year age groups. Age group 45–54 yrs.

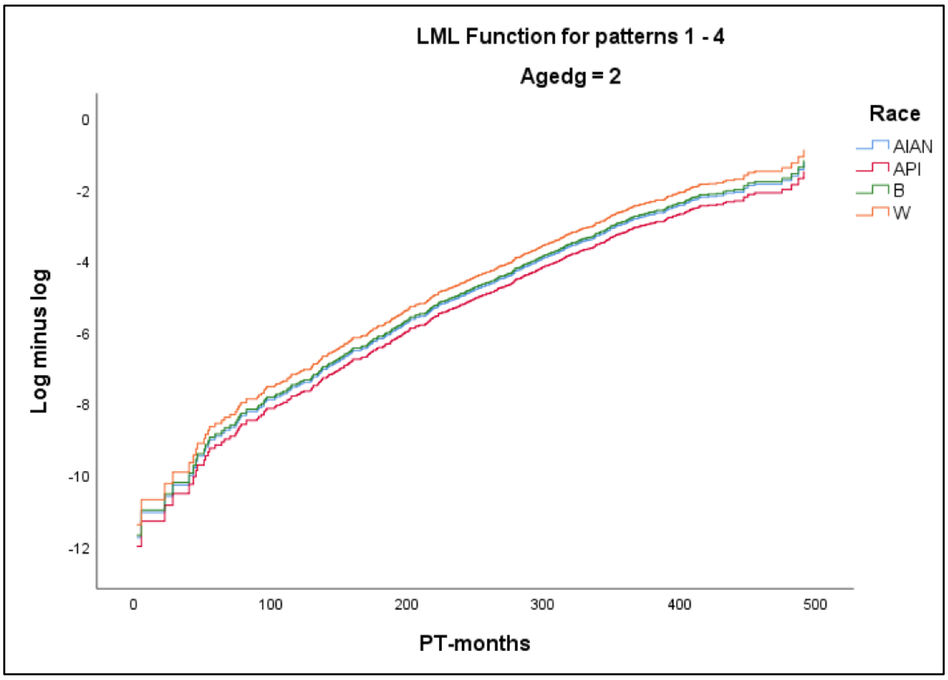

Figure S4. Log(–log survival) curves for AD death in women diagnosed with breast cancer at 45+ years. Cox model with variable race stratified on five 10-year age groups. Age group 55–64 yrs.

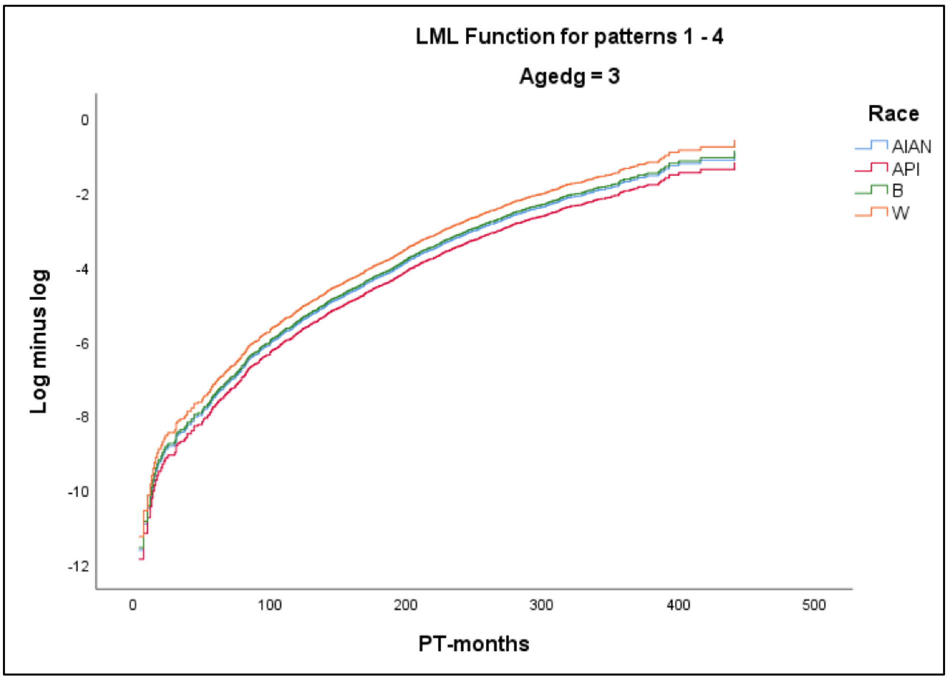

Figure S5. Log(–log survival) curves for AD death in women diagnosed with breast cancer at 45+ years. Cox model with variable race stratified on five 10-year age groups. Age group 65–74 yrs.

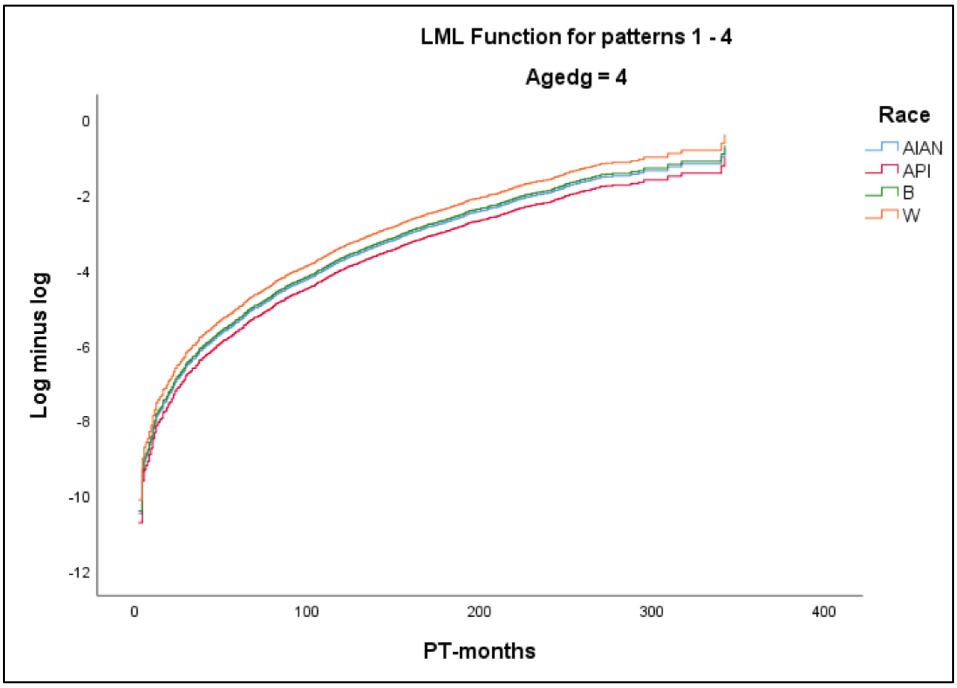

Figure S6. Log(–log survival) curves for AD death in women diagnosed with breast cancer at 45+ years. Cox model with variable race stratified on five 10-year age groups. Age group 75–84 yrs.

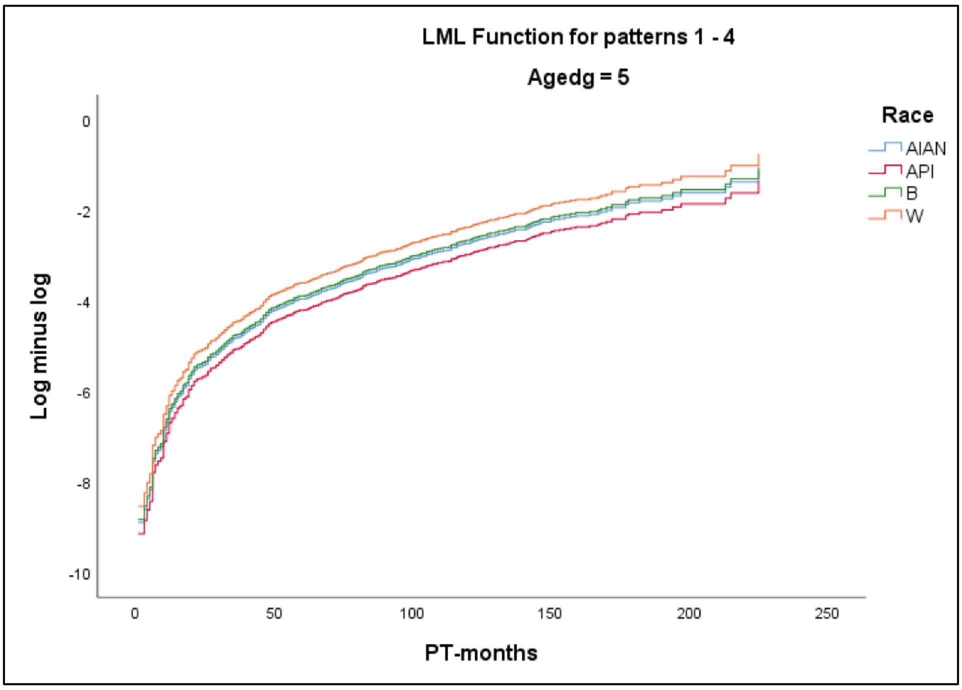

Figure S7. Log(–log survival) curves for AD death in women diagnosed with breast cancer at 45+ years. Cox model with variable race stratified on five 10-year age groups. Age group 85+ yrs.

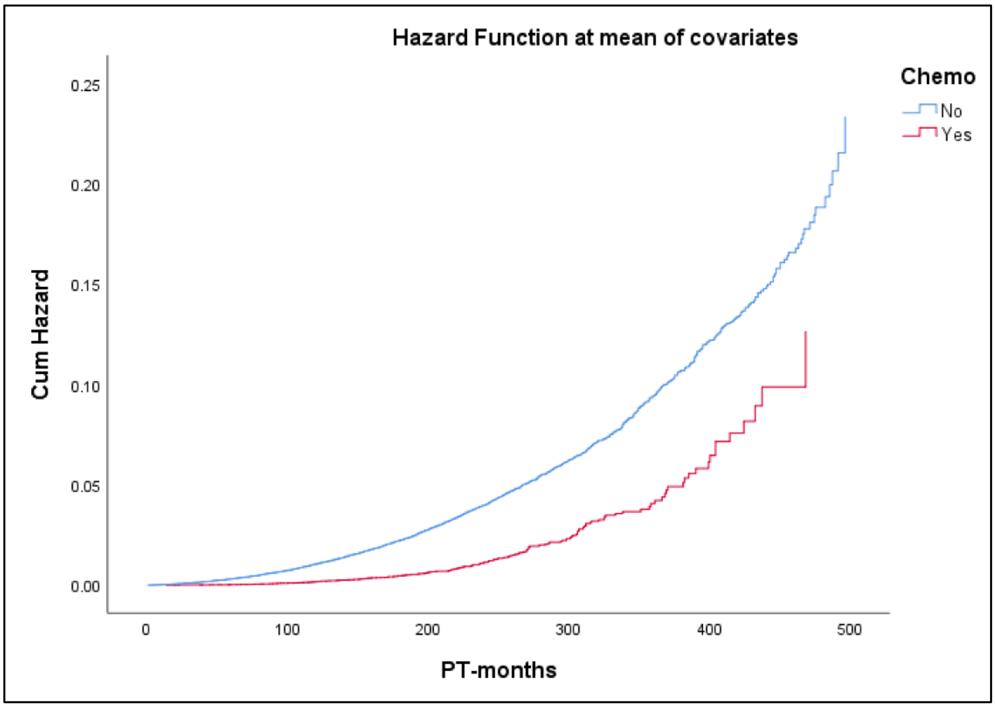

Figure S8. Baseline cumulative hazard curves for AD death in white women diagnosed with breast cancer at 45+ years. Cox model stratified on chemotherapy status.

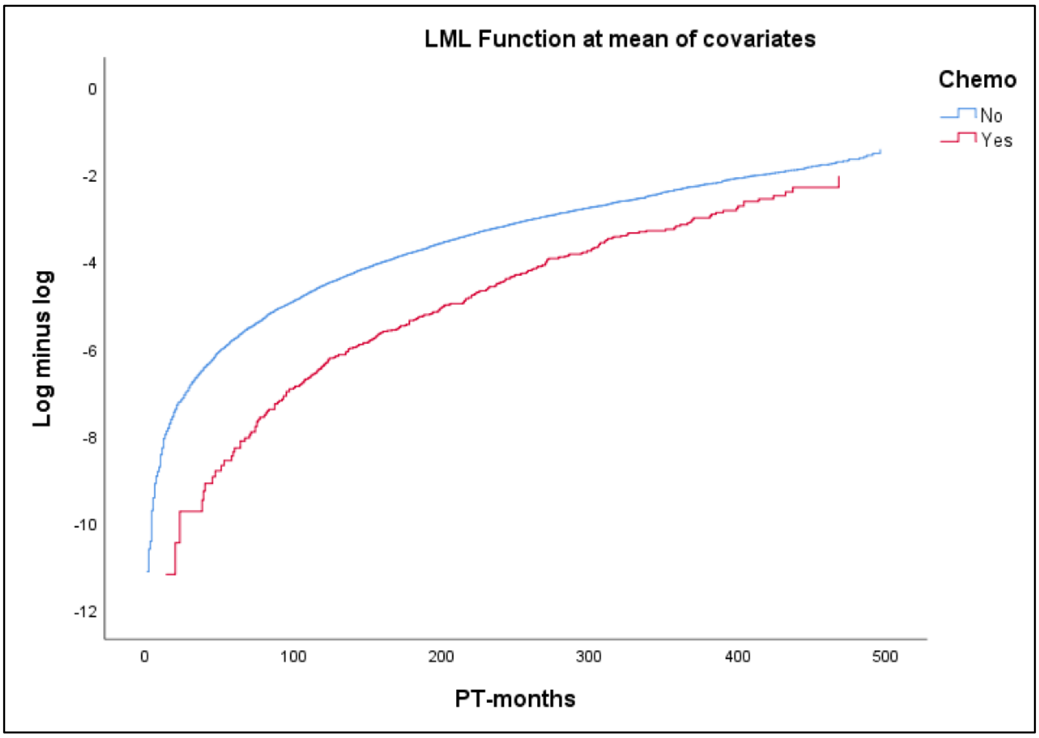

Figure S9. Log(−log survival) curves for AD death in white women diagnosed with breast cancer at 45+ years. Cox model stratified on chemotherapy status.

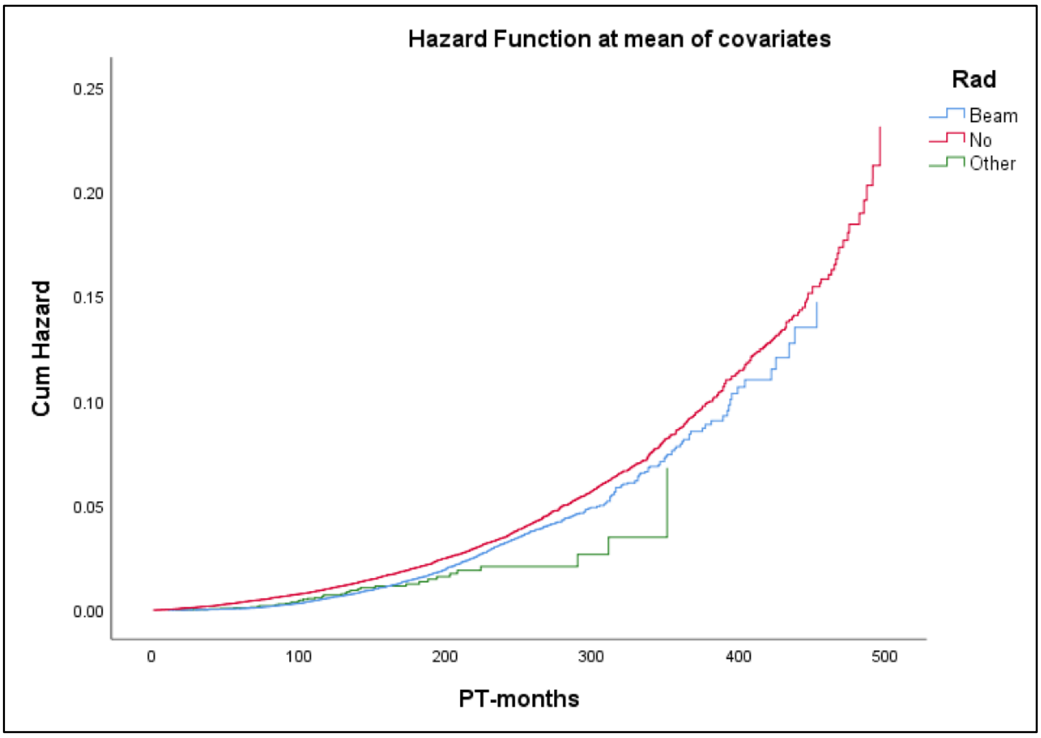

Figure S10. Baseline cumulative hazard curves for AD death in white women diagnosed with breast cancer at 45+ years. Cox model stratified on radiotherapy status.

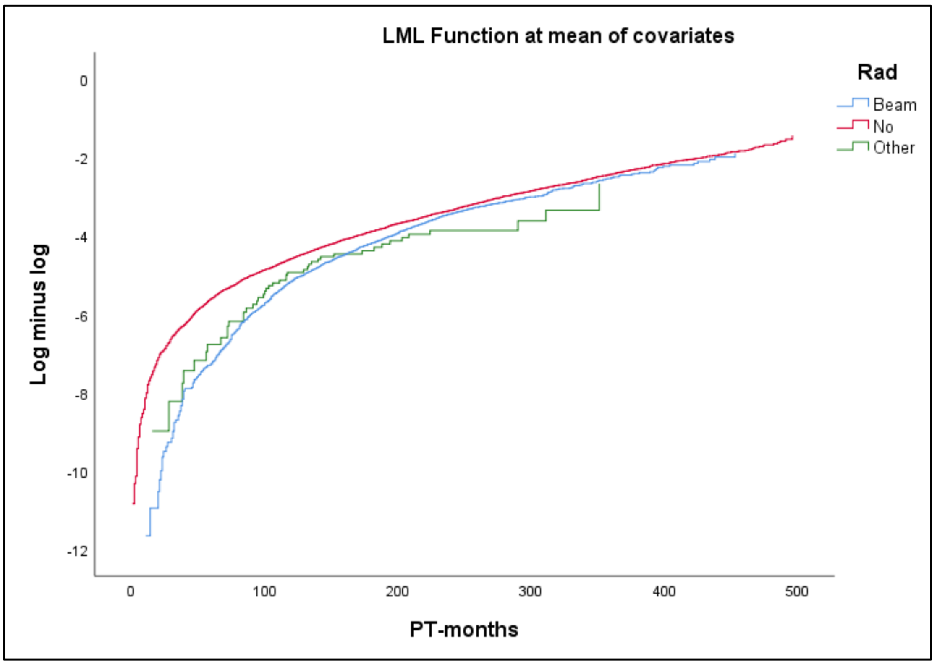

Figure S11. Log(–log survival) curves for AD death in white women diagnosed with breast cancer at 45+ years. Cox model stratified on radiotherapy status.

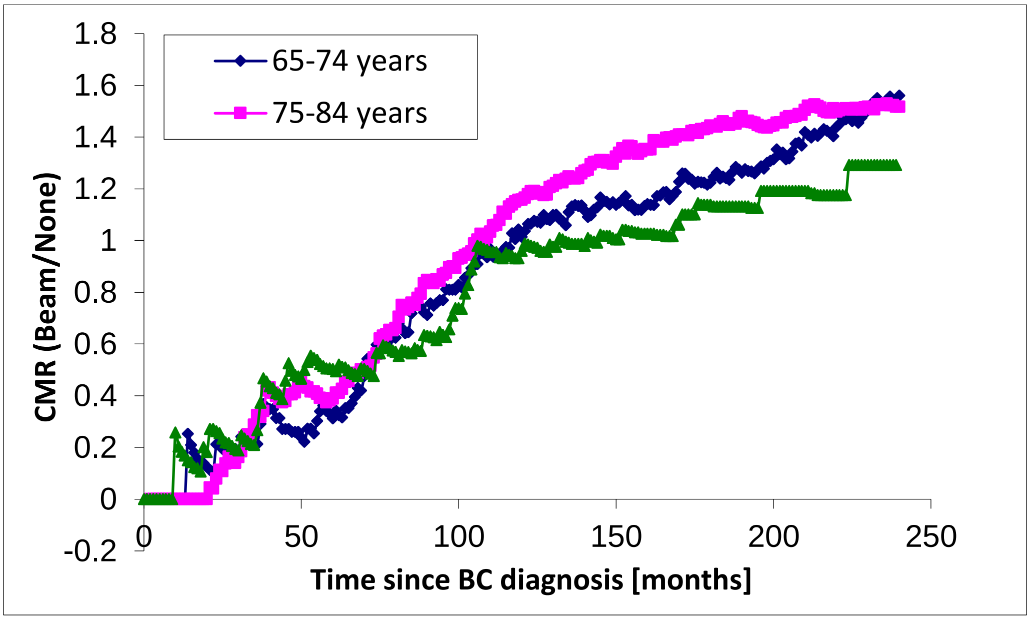

Figure S12. Cumulative AD mortality ratios (CMRs) for beam radiotherapy vs no/unknown radiation therapy groups. Point estimates shown for white female breast cancer (BC) patients diagnosed at 3 different age groups.

© 2020 by the authors. Licensee MDPI, Basel, Switzerland. This article is an open access article distributed under the terms and conditions of the Creative Commons Attribution (CC BY) license (http://creativecommons.org/licenses/by/4.0/).
